# SLC25A33-mediated mitochondrial DNA synthesis plays a critical role in the inflammatory response of M1 macrophages by contributing to mitochondrial ROS and VDAC oligomerization

**DOI:** 10.7150/ijbs.96563

**Published:** 2025-04-21

**Authors:** Daehoon Kim, Jonghwa Jin, Yu-Rim Lee, Dong-Ho Kim, Soo-Young Park, Jun-Kyu Byun, Yeon-Kyung Choi, Keun-Gyu Park

**Affiliations:** 1 Department of Biomedical Science, Kyungpook National University, Daegu 41566, Korea.; 2 Department of Internal Medicine, School of Medicine, Kyungpook National University, Kyungpook National University Hospital, Daegu 41944, Korea.; 3 Department of Internal Medicine, School of Medicine, Kyungpook National University, Kyungpook National University Chilgok Hospital, Daegu 41404, Korea.; 4 Research Institute of Pharmaceutical Sciences, College of Pharmacy, Kyungpook National University, Daegu 41566, Korea.; 5 Research Institute of Aging and Metabolism, Kyungpook National University, Daegu 41566, Korea.

## Abstract

M1 macrophage polarization is modulated by the release of mitochondrial DNA (mtDNA) and induces the inflammatory immune response, which is further increased by the generation of mitochondrial reactive oxygen species (mtROS). The pyrimidine nucleotide carrier SLC25A33 is located in the mitochondrial inner membrane and is linked to mtDNA synthesis, but its role in the M1 macrophage inflammatory immune response remains unclear. Here, we elucidate the regulatory mechanisms responsible for upregulation of SLC25A33 expression during M1 macrophage polarization, SLC25A33-mediated mtROS production, and the inflammatory response. SLC25A33 expression was significantly elevated in CD14+ monocytes derived from patients with sepsis and LPS/interferon-gamma (IFN-γ)-stimulated peritoneal macrophages (PMs). SLC25A33 was upregulated by ATF4 through the MyD88-PI3K-mTORC1 pathway in LPS/IFN-γ-stimulated PMs. Furthermore, SLC25A33 increased mtDNA synthesis and the release of mtDNA into the cytosol, which was facilitated by mtROS-mediated voltage-dependent anion channel (VDAC) oligomer formation, thereby contributing to activation of the cGAS-STING inflammatory pathway. Conversely, *SLC25A33* knockdown and pyridoxal 5'-phosphate treatment, which inhibits SLC25A33 activity, decreased mtDNA release and reduced M1 macrophage polarization and associated inflammatory responses. These findings were consistent across *in vitro* and *in vivo* sepsis models, as well as in septic patients with liver abscesses. Our findings underscore the significant role of SLC25A33 in inflammation, suggesting that targeting of SLC25A33 could be a promising therapeutic strategy for the management of M1 macrophage-mediated inflammatory diseases, including sepsis.

## Introduction

Mitochondria, while traditionally only considered cellular energy sources, also form intricate signaling hubs that regulate innate immune pathways 1, 2. Mitochondria can therefore modulate both the metabolic and physiological states of various immune cells, largely shaping the overall immune response 1, 3-5. As such, a growing body of evidence has highlighted the pivotal role of mitochondrial DNA (mtDNA) in governing key signaling cascades involved in the inflammatory response 6, 7. mtDNA can be released from the mitochondria under various pathological conditions, such as mitochondrial ROS (mtROS) production, oxidative and genotoxic stress, high pro-inflammatory cytokine expression, viral infection, and mitochondrial dysfunction 6-8. mtDNA released into the cytoplasm and extracellular environment triggers various pattern recognition receptors and innate immune responses, such as cyclic GMP-AMP synthase (cGAS)-stimulator of interferon genes (STING), TLR9, and inflammasome assembly 8, 9. Activation of these receptors results in the production of type I interferons (IFN-I) and inflammatory cytokines, thereby mounting a robust inflammatory response 10.

M1 macrophages, conventionally termed classically activated macrophages, play a pivotal role in mediating inflammatory responses, and their activation is often instigated by microbial products or inflammatory cytokines, such as interferon-gamma (IFN-γ) and Toll-like receptor (TLR) ligands 11. These differentiated macrophages produce diverse inflammatory mediators such as nitric oxide and the pro-inflammatory cytokines IL-1β, IL-6, and TNF-α, which subsequently propagate the inflammatory response 12. The intricate polarization of macrophage towards the M1 phenotype is governed by a complex regulatory network consisting of multiple signaling pathways and transcription factors 11. Particularly, mitochondrial regulation plays a crucial role in M1 macrophage polarization 13-16. mtROS has been reported to function as a critical second messenger in M1 macrophages, provoking an elevated production of pro-inflammatory cytokines 17. Recent studies have also shown that disruption of mitochondrial membrane integrity leads to the release of mitochondrial molecules, such as mtDNA, which activates inflammatory signaling in macrophages 13, 16. Nevertheless, the underlying mechanism via which mtDNA and mtROS are interconnected during M1 macrophage polarization and the subsequent inflammatory response remains incompletely understood.

The mitochondrial carrier family (solute carrier family 25, SLC25) proteins are located primarily in the inner membrane of mitochondria, and are responsible for the transport of specific molecules across the mitochondrial membrane, and therefore require strict regulation in different cells and under various physiological and pathological conditions 18. The SLC25 family is comprised of 53 mitochondrial carriers in humans, some of which have been directly or indirectly implicated in the inflammatory response 19. Notably, recent studies have demonstrated that overexpression of SLC25A33, a transporter for uracil, thymine, and cytosine (deoxy)nucleoside di- and triphosphates, results in the activation of the cGAS-STING-TBK1 signaling pathways through the release of mtDNA in both mouse retinas and cultured cells 20, 21. However, the relevance of SLC25A33 in macrophages within a clinical setting and the regulatory pathways governing SLC25A33 expression remain unclear. Furthermore, the role of SLC25A33-mediated mtDNA synthesis and mtROS production in facilitating mtDNA release during macrophage polarization and the dysregulated inflammatory response warrants further investigation.

In this study, we characterized the regulatory mechanism controlling SLC25A33 expression and its involvement in macrophage polarization. We also determined whether downregulation of SLC25A33 could modulate M1 polarization of macrophages and limit dysregulated inflammatory responses and the severity of sepsis.

## Materials and Methods

### Isolation of peritoneal macrophages

Peritoneal macrophages (PMs) were isolated from 7-8-week-old male C57BL/6 mice (DooYeol Biotech, Seoul, South Korea). Mice received an intraperitoneal (i.p.) injection of 3% thioglycolate media and were sacrificed 4 days later. Phosphate Buffered Saline (PBS) was subsequently injected into the peritoneum, retrieved, and dispensed into a tube on ice. PMs were collected by centrifugation. All animal procedures received approval from the Institutional Animal Care and Use Committee (IACUC) of Kyungpook National University (approval number: 2023-0328, 2023-0582).

### Isolation of human PBMCs and CD14^+^ monocytes

Human peripheral blood mononuclear cells (PBMCs) were isolated from whole blood collected from healthy controls and septic patients with liver abscesses. Details of the characteristics of the controls and patients can be found in Table S3-4. Blood samples from septic individuals were obtained within 24 h of diagnosis. Collection was conducted after obtaining written, informed consent and following approval from the Kyungpook National University Chilgok Hospital (IRB No. KNUH 2020-01-005). Isolation of PBMCs was performed using SepMate™ (STEMCELL Technologies, Vancouver, Canada) through density gradient centrifugation. CD14+ monocytes were then separated from the PBMCs using the Pan Monocyte Isolation Kit with LS columns (Miltenyi Biotec, Bergisch Gladbach, Germany), according to the manufacturer's instructions.

### Cell culture and chemical treatments

Primary PMs were maintained in RPMI 1640 Medium. To induce M1 polarization, PMs were treated with LPS (100 ng/mL) (Sigma, St. Louis, MO, USA) and IFN-γ (10 ng/mL) (R&D Systems Inc., St. Paul, MN, USA) for 24 h. Where indicated, cells were treated with PLP (400 μM; Sigma), ST2825 (10 μM; MedChemExpress, Monmouth Junction, NJ, USA), MRT67307 (1 µM; Selleckchem, Houston, TX, USA), LY294002 (25 nM; Abcam, Cambridge, UK), Takinib (10 μM; MedChemExpress), Rapamycin (50 nM; Sigma), 2',3'-Dideoxycytidine (40 μM; Thermo Fisher Scientific, Waltham, MA, USA), VBIT-4 (10 μM; Selleckchem), and MitoTEMPO (500 μM; Sigma).

### Library preparation and sequencing

For control and test RNA, library construction was performed using the QuantSeq 3' mRNA-Seq Library Prep Kit (Lexogen, Inc., Austria) according to the manufacturer's instructions. Briefly, 500 ng of total RNA was hybridized with an oligo-dT primer containing an Illumina-compatible sequence at its 5' end and reverse transcribed. After RNA template degradation, second-strand synthesis was initiated with a random primer that included an Illumina-compatible linker sequence at its 5' end. The double-stranded library was then purified using magnetic beads to remove all reaction components. Amplification of the library was performed to add the complete adapter sequences required for cluster generation, and the finished library was subsequently purified from PCR components. High-throughput single-end 75 bp sequencing was carried out using the NextSeq 500 system (Illumina, Inc., USA).

### Data analysis

QuantSeq 3' mRNA-Seq reads were aligned using Bowtie2 22. Bowtie2 indices were generated from either the genome assembly sequence or the representative transcript sequences by alignment with the genome and transcriptome, respectively. The resulting alignment file was used to assemble transcripts, estimate their abundance, and detect differential gene expression. Differentially expressed genes were determined based on counts from unique and multiple alignments using coverage in Bedtools 23. The read count (RC) data were processed using the quantile normalization method with EdgeR within R (R: a language and environment for statistical computing. R Foundation for Statistical Computing, 2016) and Bioconductor 24. Pathway analysis was conducted to identify significantly enriched biological pathways among the differentially expressed genes. The Kyoto Encyclopedia of Genes and Genomes (KEGG) database was used to match these genes to known inflammatory pathways. Enrichment analysis was performed using the online Database for Annotation, Visualization, and Integrated Discovery (DAVID) tool (https://david.ncifcrf.gov/), which provides gene annotation and integrated discovery functions. The analysis utilized the default parameters, with a false discovery rate threshold of 0.05 set to determine statistical significance.

### Quantitative PCR

Total RNA was extracted using the AccuPrep Universal RNA Extraction Kit (Bioneer, Seoul, South Korea). cDNA was synthesized with the RevertAid First Strand cDNA Synthesis Kit (Thermo Fisher Scientific, Inc.). For the measurement of cytosolic mtDNA, the cytosolic fraction was extracted using the Mitochondria/Cytosol Fraction Kit (Abcam). Gene expression analysis was performed using the QuantStudio 5 (Applied Biosystems, Foster City, CA, USA) with SYBR Green PCR Master Mix (Applied Biosystems). Gene expression levels were normalized to the corresponding levels of mouse 36B4, mouse β-actin, or human STX5A mRNA. The primer sequences used in this study were as follows: 36B4 forward, ACCTCCTTCTTCCAGGCTTT, and reverse, CTCCAGTCTTTATCAGCTGC; iNOS forward, GGCAGCCTGTGAGACCTTTG, and reverse, TGCATTGGAAGTGAAGCGTTT; SLC25A33 forward, ATCGTCTCCTCCACAGATGG, and reverse, ATGACCTCGTGTGGGTAAGC; TNF-α forward, AGCCGATGGGTTGTACCTTG, and reverse, ATAGCAAATCGGCTGACGGT; IL-6 forward, TTCTCTGGGAAATCGTGGAAA, and reverse, TGCAAGTGCATCATCGTTGTT; IL-1β forward, GAGCACCTTCTTTTCCTTCATCTT, and reverse, TCACACACCAGCAGGTTATCATC; and ATF4 forward, TGAACCCAATTGGCCATCTC, and reverse, GGGAAAGGCTGCAAGAATGTAA. The primer sequences used to measure mtDNA copy number were as follows: β-actin forward, CATTGCTGACAGGATGCAGAAGG, and reverse, TGCTGGAAGGTGGACAGTGAGG; and CytB forward, GCTTTCCACTTCATCTTACCATTTA, and reverse, TGTTGGGTTGTTTGATCCTG. The primer sequences used for amplification of human CD14+ monocyte RNA were as follows: STX5A forward, GAACACGGATCAGGGTGTCTA, and reverse, ACGTTCTCGTCGTCGATCCTCTG; SLC25A33 forward, GGTGTGGAGGCACAGTTGGT, and reverse, CTTGAAGACTGCAACCGTGTCT; TNF-α forward, GGAGAAGGGTGACCGACTCA, and reverse, CAGACTCGGCAAAGTCGAGAT; IL-6 forward, AGCTGCAGGCACAGAACCA, and reverse, AGCTGCGCAGAATGAGATGA; and IL-1β forward, CGAATCTCCGACCACCACTAC, and reverse, TCCATGGCCACAACAACTGA.

### Western blot analysis

Cell lysates were separated by NuPAGE 4-12% gels (Thermo Fisher Scientific) or Tris-Glycine gels and transferred onto PVDF membranes (Millipore Corporation, Bedford, MA, USA). After blocking with 5% skimmed milk, the membranes were incubated with antibodies against the following proteins: iNOS (BD Biosciences, San Jose, CA, USA), CD80, CD86, PI3K, p-PI3K (Tyr458), p70S6K, p-p70S6K (T389), STING, p-STING (Ser365), p65, p-p65 (Ser536), ATF4, IRF3, p-IRF3 (Ser396), IKKβ, p-IKKα/β (T176/180), IκBα, p-IκBα (S32), p-IκBα (S32/36), p-TAK1 (T184/187), AKT, p-AKT (Ser473), mTOR, MyD88, TBK1 (Cell Signaling Technology, Danvers, MA, USA), TAK1 (ABclonal Technology, Boston, MA, USA), and β-Actin (Sigma). Following washes in TBST, the membranes were visualized with a goat anti-IgG secondary antibody (GeneTex, Irvine, CA, USA).

### Protein oligomerization assay

To investigate VDAC1 oligomerization, PMs treated with LPS (100 ng/mL) and IFN-γ (20 ng/mL) were washed with Dulbecco's Phosphate Buffered Saline, harvested, and subjected to crosslinking with 200 μM EGS (ethylene glycol bis(succinimidyl succinate), Sigma) in PBS (pH 8.3) for 20 minutes at 30 °C. The samples were then analyzed by NuPAGE 4-12% (Thermo Fisher Scientific) gradient gel electrophoresis followed by immunoblotting with an anti-VDAC1 antibody (Cell Signaling Technology).

### Measurement of cytokine secretion

PMs were treated with or without LPS/IFN-γ for 24 h, after which cytokines in the culture media were measured using ELISA MAX™ Deluxe Set Mouse TNF-α, IL-6, and IL-1β kits (BioLegend, San Diego, CA, USA) according to the manufacturer's instructions. Data were normalized to the protein concentration of each sample.

### Measurement of mitochondrial reactive oxygen species

mtROS was assessed using MitoSOX™ Red mitochondrial superoxide indicator (Invitrogen, Waltham, MA, USA). PMs were treated with 5 μM MitoSOX reagent working solution and incubated for 15 minutes at 37℃ and 5% CO_2_ in the dark. After cells were washed with HBSS buffer, cells were stained with NucBlue Live Cell Stain Ready Probes (Invitrogen). MitoSOX fluorescence intensity were quantified using Image J software.

### Measurement of luciferase activity

Cells were infected with a lentivirus containing the reporter gene and Gaussia luciferase (GeneCopoeia, Rockville, MD, USA). After 24 h of LPS treatment, the medium was harvested and luciferase activity was measured using the Secrete-Pair Dual Luminescence Assay Kit (GeneCopoeia).

### Immunofluorescence

PMs were incubated with MitoTracker™ Red CMXRos (Invitrogen) before fixation with 4% paraformaldehyde for 30 minutes at room temperature. Subsequently, samples were permeabilized with 0.5% Triton X-100 for 15 minutes. After blocking with 5% bovine serum albumin for 40 minutes, the cells were incubated overnight at 4°C with anti-dsDNA antibody (Abcam) or anti-DNA antibody (PROGEN, Heidelberg, Germany). Following washing, the cells were incubated with Alexa Fluor® 488 or Fluor® 568-conjugated secondary antibodies (Invitrogen) for 1 h. To assess mtDNA replication in primary macrophages, the cells were pre-treated with 10 μM PdG (6-O-Propynyl-dG) (Jena Bioscience, Jena, Germany) for 1 h before LPS/ IFN-γ treatment. For PdG detection, Alexa Fluor® 647-Picolyl-Azide was used in combination with the CuAAC Cell Reaction Buffer Kit (BTTAA-based) (Jena Bioscience) according to the manufacturer's instructions.

### Immunohistochemistry

Lung and intestinal tissue samples were fixed with 4% paraformaldehyde and embedded in paraffin. Antigen retrieval was performed using the IHC-Tek Epitope Retrieval Steamer Set (IHC World, Ellicott, MD, USA) after deparaffinizing the section. Endogenous peroxidase was blocked by incubating with 3% H_2_O_2_ for 10 minutes. The slides were then incubated overnight at 4℃ with mouse anti-CD86 (Cell Signaling Technology). Detection was achieved using the UltraVision LP Detection System (Thermo Fisher Scientific) or HRP-conjugated secondary antibodies (Abcam) with 3,3'-diaminobenzidine as the chromogen. Nuclei were counterstained with Dako hematoxylin (Agilent, Santa Clara, CA, USA). For fluorescence staining of tissues, the slides were incubated overnight at 4℃ with antibodies against CD80 (Santa Cruz Biotechnology, Santacruz, CA, USA) and SLC25A33 (Origene, MD, Rockville, USA). After washing with PBS, the slides were incubated for 1 h at room temperature with Alexa Fluor 488-conjugated IgG and Alexa Fluor 568-conjugated IgG antibodies. Nuclei were stained with DAPI (Vector Laboratories, Burlingame, CA, US1A). The results were graded as negative (0), low positive (1), and high positive (3) by an experienced pathologist, following a previously established procedure 25.

### Electrophoretic mobility shift assay (EMSA)

Nuclear extracts were prepared using the Nuclear Extraction Kit (Abcam). EMSA assay was performed using the NFκB EMSA Kit (Signosis, Santa Clara, CA, USA). Briefly, the nuclear extract (5 μg) was incubated with poly d(I-C), 5X binding buffer, and the biotin-labeled NF-κB transcription probe at 22℃ for 30 minutes in a PCR machine. Then, samples were loaded onto 6.5% non-denaturing polyacrylamide gel. The proteins were transferred to an NB Membrane (Signosis) and immobilized by exposure to UV light at 120,000 μJ/cm² for 1 minute. The membrane was then incubated with Streptavidin-HRP Conjugate (Signosis) to detect biotinylated proteins.

### *In vitro* kinase assay for IKKβ

Whole-cell lysates were prepared form PMs, and immunoprecipitated with an anti-IKKβ antibody (Cell Signaling). The IKKβ activity in each group was measured using the ADP-Glo^TM^ Kinase Assay Kit (Promega, Madison, WI, USA) with recombinant IκBα protein (Applied Biological Materials, Richmond, BC, Canada) as the substrate.

### Chromatin immunoprecipitation (ChIP)

The ChIP assay was performed in accordance with the manufacturer's protocol (SimpleChIP Enzymatic Chromatin IP Kit Agarose Beads, Cell Signaling). Each ChIP experiment was conducted with 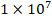
cells and anti-ATF4 antibody (Cell Signaling). Rabbit IgG was employed as a control for non-specific DNA immunoprecipitation. For PCR amplification, the immunoprecipitated DNA was amplified for the *SLC25A33* promoter region using forward (5′- GCAGGCTGTTTAGGTGACGT-3′) and reverse (5′-TACCCTGAGCTCCCGATCTT-3′) primers. The PRL30 primers provided with the kit were utilized as a control.

### Murine sepsis model

To evaluate the protective effects of pyridoxal 5'-phosphate hydrate in an LPS-induced sepsis model, C57BL/6 mice were i.p. injected with 20 mg/kg of pyridoxal 5'-phosphate hydrate (Sigma), 2 h prior to challenge with 30 mg/kg of LPS (Sigma). The cecal ligation and puncture (CLP) model was established in line with previous work 26. Briefly, mice were firstly anesthetized with isoflurane. After making a 1 cm midline abdominal incision, the cecum was exposed and its length was ligated at approximately two-thirds from the distal tip to maintain intestinal continuity. Subsequently, two punctures were made on the cecum using a 22-gauge needle, and a small amount of its contents was expressed through these punctures. As a control group, a sham operation involving laparotomy and exposure of the cecum without further manipulation was performed. The incision was then closed, and subcutaneous administration of 1 mL of normal saline was carried out. Following the surgery, the animals were allowed unrestricted access to food and water. Samples were collected from the surviving mice and those sacrificed at specific time points for analysis.

### Generation of bone marrow-derived macrophages (BMDMs)

BMDMs were isolated from femurs and tibias by passing the marrow through a 70 μm nylon mesh filter to remove debris. Cells were subsequently cultured in RPMI 1640 with 10% FBS (HyClone Laboratories, Utah, USA), 1% penicillin/streptomycin (Gibco, New York, USA) and M-CSF (R&D Systems, Minneapolis, USA) for 6 days in culture.

### siRNA transfection and lentiviral transduction

Cells were transfected with scramble siRNA, siSLC25A33, siMyD88, siPI3K, simTOR, siTAK1, siTBK1, siATF4 (Bioneer), SLC25A33 ORF Clone (Origene) or ATF4 ORF Clone (GeneCopoeia) using Lipofectamine^TM^ 3000 (Thermo Fisher Scientific). Lentiviral plasmid vectors shSLC25A33, shATF4, and control shRNA (Origene) were transduced into HEK293T cells using the Lenti-X Packaging Single Shot system (Takara Co., Tokyo, Japan) for 48 h, and the supernatants were collected and stored at -80℃. BMDMs were transduced with the lentiviral plasmid vectors in the presence of 6 µg/mL polybrene.

### Macrophage depletion and reconstitution

Macrophages were depleted *in vivo* using clodronate liposomes. 6-week-old male C57BL/6 mice were injected intravenously through the retro-orbital venous sinus with clodronate liposomes and control liposomes (200 μL) (SamboMedical, Gyeonggi-do, South Korea). Two days later, the presence of F4/80+ macrophages in the spleen were confirmed by FACS. Macrophage-depleted mice were then reconstituted with 2×10^6^ transduced BMDMs through intravenous injection of the BMDMs in 200 μL of PBS.

### Statistical analysis

Data are presented as the average ± standard error of the mean (SEM) from a minimum of three independent experiments. Statistical significance was determined using Student's t-test (Prism software), and p < 0.05 was considered statistically significant.

## Results

### SLC25A33 is upregulated in LPS/IFN-γ-treated M1 macrophages and monocytes from patients with sepsis

**Figure 1:**
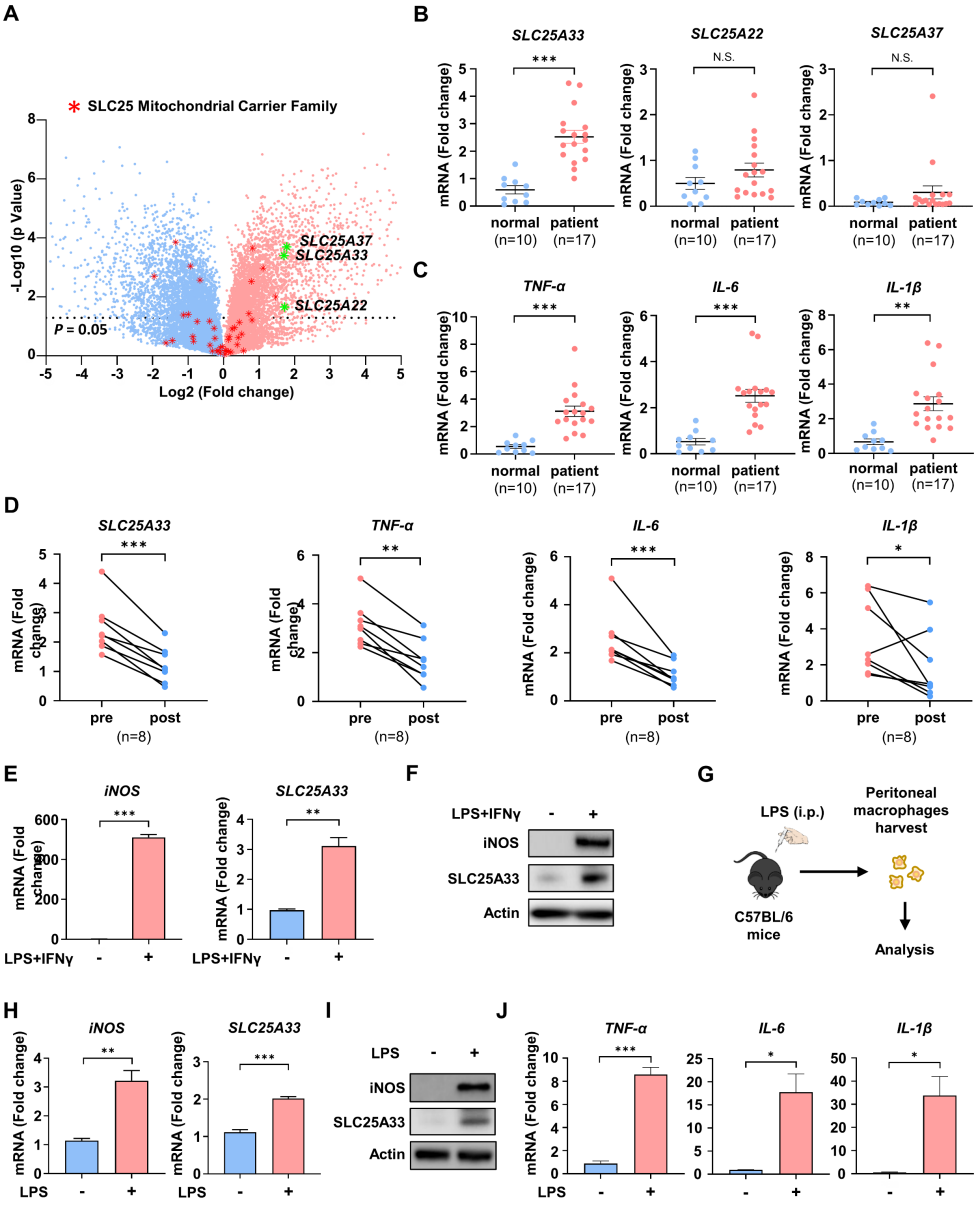
** SLC25A33 is upregulated in LPS/IFN-γ-induced M1 macrophages and CD14+ monocytes from septic patients. (A)** Volcano plot illustrating gene expression differences between untreated PMs and LPS/IFN-γ-treated PMs. Red asterisks denote differentially expressed genes in the *SLC25* family, and green asterisks highlight highly upregulated genes within the same family. **(B-D)** Relative mRNA expression of *SLC25A33*, *SLC25A22*, and *SLC25A37* (B) and pro-inflammatory cytokines (C) in CD14+ monocytes from septic patients with liver abscesses (n=17) compared with healthy controls (n=10), and (D) changes in relative mRNA expression pre- and post-recovery in these septic patients. **(E-F)** Relative *iNOS* and *SLC25A33* mRNA expression (E) and their protein levels (F) in LPS/IFN-γ-treated PMs. **(G)** An *ex vivo* experimental protocol for the isolation and harvesting of PMs from C57BL/6 mice treated with LPS. **(H-J)** Relative mRNA expression of *iNOS* and *SLC25A33* (H), their corresponding protein expression levels (I), and relative mRNA expression of pro-inflammatory cytokines (J) in PMs isolated from mice following intraperitoneal LPS injection. In all *in vitro* experiments, PMs were treated with LPS (100 ng/mL) and IFN-γ (10 ng/mL) for 24 h. For *ex vivo* experiments, mice were injected intraperitoneally with LPS (2 mg/kg) and PMs were isolated after 24 h. All experimental data were verified in at least three independent experiments. Data are presented as the mean ± SEM. N.S., not significant, *p < 0.05, **p < 0.01 and ***p < 0.001. iNOS: inducible nitric oxide synthase; PMs: peritoneal macrophages.

To assess differences in gene expression profile responses to LPS/IFN-γ, we conducted mRNA sequencing (mRNA-seq) on LPS/IFN-γ-treated PMs. KEGG pathway enrichment analysis identified up- and down-regulated inflammatory response pathways (Fig. S1A) and significant enrichment in metabolic pathways, as well as in pathways related to cancer, immune response, and inflammation, including the TNF signaling and NF-κB signaling pathways. Comprehensive analysis of gene expression identified the top 1,000 most significantly up- and down-regulated genes (Table S1). Notably, members of the mitochondrial carrier family were among those whose expression was affected in LPS/IFN-γ-treated PMs. To further investigate the involvement of mitochondrial carriers in M1 macrophage polarization, we determined the gene expression levels of SLC25 family proteins in LPS/IFN-γ-treated PMs by mRNA-seq. Our analysis revealed a marked increase in the expression of *SLC25A22*, *SLC25A33*, and *SLC25A37* in PMs stimulated with LPS/IFN-γ compared with unstimulated PMs (Fig. 1A). Subsequently, we measured the mRNA expression of *SLC25A22, SLC25A33*, and *SLC25A37*, along with that of pro-inflammatory cytokines, including *TNF-α, IL-6*, and *IL-1β*, in CD14+ monocytes from sepsis patients with liver abscesses. Compared with healthy controls, patient-derived monocytes showed significantly increased expression of *SLC25A33* and pro-inflammatory cytokines, while *SLC25A22* and *SLC25A37* did not show significant changes in expression (Fig. 1B and C). The increased expression of *SLC25A33* was further corroborated by data from Digital Gene Expression analysis (Table S2). We then performed correlation analyses between *SLC25A33* expression and inflammatory markers in the patient samples. The results revealed significant positive correlations between *SLC25A33* expression and the expression of pro-inflammatory cytokines *TNF*-α and *IL-6* in the pre-treatment data (Fig. S1B). Although *IL-1β* did not reach statistical significance, its expression tended to correlate with *SLC25A33* expression. Notably, the elevated expression of *SLC25A33* and cytokines decreased after the patients recovered from sepsis (Fig. 1D). Consistent with our mRNA-seq data, we further validated the upregulation of *SLC25A33* mRNA expression and protein levels in LPS/IFN-γ-treated macrophages, along with inducible nitric oxide synthase (iNOS), a recognized marker for M1 macrophages (Fig. 1E and F). Analysis of isolated PMs from mice post LPS injection (Fig. 1G) demonstrated a similar increase in mRNA and protein expression of iNOS and SLC25A33 in LPS/IFN-γ-treated macrophages (Fig. 1H-I), in addition to increased mRNA expression of the pro-inflammatory cytokines *TNF-α*, *IL-6*, and *IL-1β* (Fig. 1J). Together, these data demonstrate a significant upregulation of SLC25A33 in LPS/IFN-γ-stimulated macrophages and CD14+ monocytes from human sepsis, suggesting a potential role for SLC25A33 in dysregulated inflammatory responses.

### Inhibition of SLC25A33 attenuates the inflammatory response and mtROS generation by M1 macrophages

**Figure 2:**
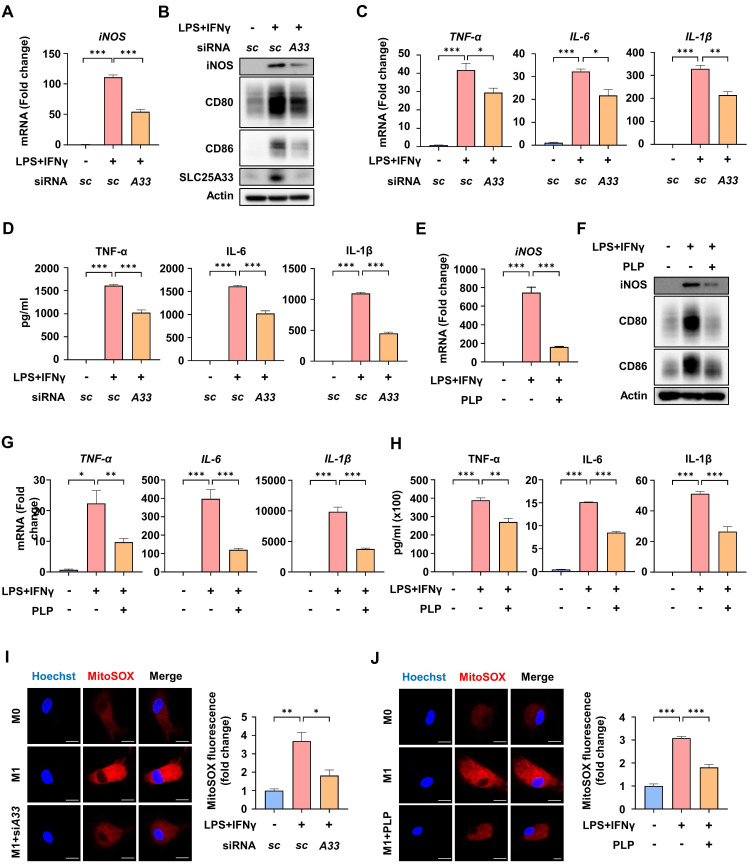
** SLC25A33 inhibition reduces LPS/IFN-γ-induced inflammatory response and mtROS of M1 macrophages. (A-B)** Effect of *SLC25A33*-targeting siRNA on *iNOS* mRNA expression (A) and protein levels of iNOS, CD80, and CD86 (B) in LPS/IFN-γ-treated PMs. **(C-D)** Effect of *SLC25A33*-targeting siRNA on relative mRNA expression (C) and secretion (D) of pro-inflammatory cytokines in the LPS/IFN-γ-treated PMs. **(E-F)** Effect of PLP on *iNOS* mRNA expression (E) and protein levels of iNOS, CD80, and CD86 (F) in the LPS/IFN-γ-treated PMs. **(G-H)** Effect of PLP on the relative mRNA expression (G) and secretion (H) of pro-inflammatory cytokines in the PMs treated with LPS/IFN-γ. **(I-J)** Representative image of immunofluorescence staining for MitoSOX Red (left panel) and quantification of fluorescence intensity (right panel) in the LPS/IFN-γ-treated PMs following treatment with *SLC25A33*-targeting siRNA (I) and PLP (J). In all experiments, PMs were treated with LPS (100 ng/mL) and IFN-γ (10 ng/mL) for 24 h, with PLP (400 μM, 24 h), MitoSOX Red (5 μM, 15 minutes), and *SLC25A33*-targeting siRNA (40 pM; 48 h). All experimental data were verified in at least three independent experiments. Data are presented as the mean ± SEM. Scale bar represents 10 μm. *p < 0.05, **p < 0.01, and ***p < 0.001. iNOS: inducible nitric oxide synthase; PMs: peritoneal macrophages; PLP: pyridoxal 5'-phosphate.

To characterize the role of SLC25A33 in the inflammatory response of M1 macrophages, we conducted knockdown experiments. *SLC25A33* knockdown significantly reduced LPS/IFN-γ-induced mRNA expression of *iNOS* and the protein levels of M1 markers such as iNOS, CD80, and CD86, without compromising cell viability (Fig. 2A-B, S2A-B). We also confirmed a decrease in iNOS protein levels following *SLC25A33* knockdown in CD14+ monocytes isolated from the septic patients (Fig. S2C). Furthermore, *SLC25A33* knockdown substantially attenuated the LPS/IFN-γ-induced increase in mRNA expression and secretion of pro-inflammatory cytokines, including TNF-α, IL-6, and IL-1β (Fig. 2C-D). Previous research has established that pyridoxal 5'-phosphate (PLP), the active form of vitamin B6, effectively inhibits activity of SLC25A33 20. Consistent with the results of the knockdown experiments, PLP treatment led to a reduction in the LPS/IFN-γ-induced upregulation of iNOS at both the mRNA and protein levels, as well as a decrease in the protein levels of other M1 markers (Fig. 2E-F), and the mRNA expression and secretion of the pro-inflammatory cytokines TNF-α, IL-6, and IL-1β without compromising cell viability (Fig. 2G-H and S2D). Interestingly, both *SLC25A33* knockdown and PLP treatment resulted in a decrease of LPS/IFN-γ-induced mtROS, as determined by MitoSOX Red staining (Fig. 2I-J). These findings suggest that SLC25A33 plays a crucial role in the inflammatory response and in increasing mtROS levels of M1 macrophages.

### The MyD88-PI3K-mTORC1 signaling pathway mediates expression of SLC25A33 in M1 macrophages via upregulation of activating transcription factor 4

**Figure 3:**
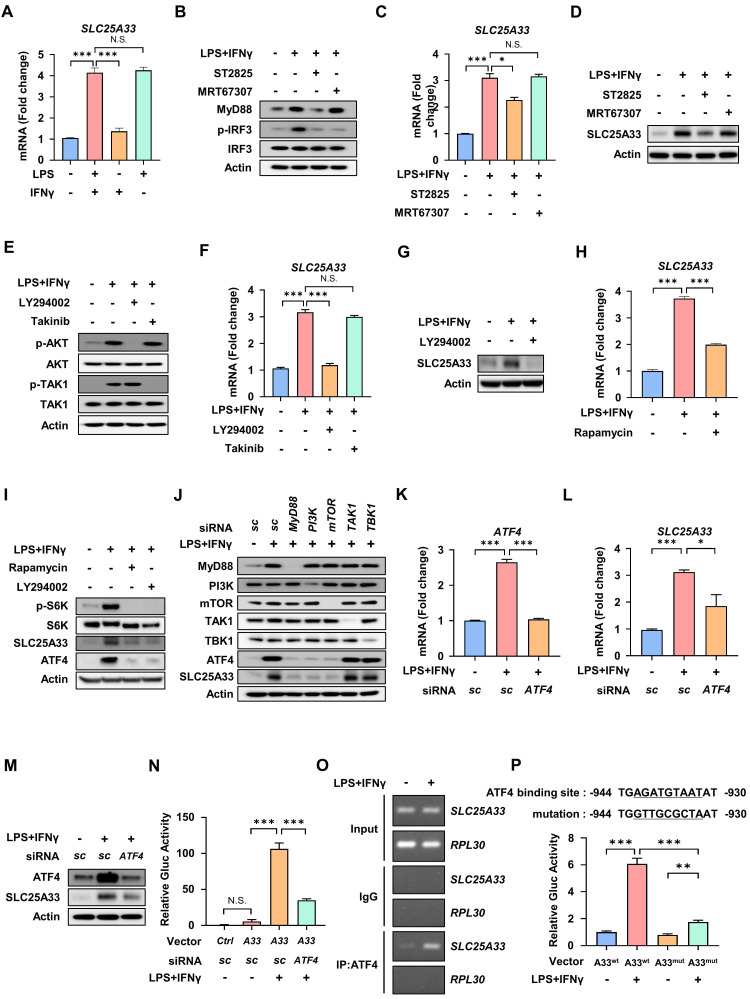
** MyD88-PI3K-mTORC1 signaling and ATF4 are essential drivers of SLC25A33 expression in M1 macrophages. (A)** Relative mRNA expression of *SLC25A33* in PMs treated with LPS, IFN-γ, or their combination.** (B-D)** Effect of the MyD88 inhibitor ST2825 and the TBK1 inhibitor MRT67307 on the levels of indicated proteins (B), as well as on relative mRNA expression (C) and protein levels (D) of SLC25A33 in the LPS/IFN-γ-treated PMs.** (E-G)** Effect of the PI3K inhibitor LY294002 or the TAK1 inhibitor Takinib on the levels of the indicated proteins (E) and their relative mRNA expression (F) and the protein levels (G, LY294002 only) of SLC25A33 in the LPS/IFN-γ-treated PMs. **(H)** Effect of rapamycin on the relative mRNA expression of *SLC25A33* in the LPS/IFN-γ-treated PMs. **(I)** Effect of rapamycin or LY294002 on protein levels of phosphorylated-S6K, S6K, SLC25A33, and ATF4 in the LPS/IFN-γ -treated PMs. **(J)** Effect of *MyD88*, *PI3K*, *mTOR*, *TAK1* or *TBK1*-targeting siRNAs on protein levels of ATF4 and SLC25A33 in the LPS/IFN-γ-treated PMs. **(K-M)** Effect of *ATF4*-targeting siRNA on the relative mRNA expression of *ATF4* (K), *SLC25A33* (L) and protein levels of ATF4 and SLC25A33 in the LPS/IFN-γ-treated PMs (M). **(N)** Effect of *ATF4*-targeting siRNA on the relative luciferase activity of the Gluc reporter in the LPS/IFN-γ-treated PMs. **(O)** ChIP assay of ATF4 binding to its putative site within the *SLC25A33* promoter in LPS/IFN-γ-treated PMs. **(P)** Effect of mutation of the putative ATF4 binding site within the *SLC25A33* promoter on the relative luciferase activity of the Gluc reporter in the LPS/IFN-γ-treated PMs. In all experiments, PMs were treated with LPS (100 ng/mL) and IFN-γ (10 ng/mL) for 24 h, with ST2825 (10 μM, 24 h), MRT67307 (1 μM, 24 h), LY294002 (25 nM, 24 h), Takinib (10 μM, 24 h), rapamycin (50 nM, 24 h), and siRNAs against each gene (40 pM, 48 h). All experimental data were verified in at least three independent experiments. Data are presented as the mean ± SEM. N.S., not significant, *p < 0.05, **p < 0.01, and ***p < 0.001. ATF4: activating transcription factor 4; Gluc: Gaussia luciferase; PMs: peritoneal macrophages.

To identify the specific signaling pathway responsible for induction of SLC25A33 in M1 macrophages, we compared the mRNA expression of *SLC25A33* in PMs treated with LPS and/or IFN-γ. Our results showed that LPS treatment alone is sufficient to significantly increase *SLC25A33* mRNA expression, while IFN-γ alone did not have a substantial effect (Fig. 3A). Upon activation of TLR4 by LPS, two crucial adaptor proteins, MyD88 and TRIF, facilitate downstream signaling 27. We therefore examined signaling downstream of LPS/TLR4 activation, focusing on the MyD88-dependent pathway that includes PI3K and TAK1 signaling, and the TRIF-dependent pathway 28. These data reveal that the MyD88 inhibitor ST2825 reduces elevated mRNA and protein expression of SLC25A33 in LPS/IFN-γ-treated PMs, suggesting that MyD88 plays a role in regulating SLC25A33 expression in M1 macrophages (Fig. 3B-D). By contrast, MRT67307, an inhibitor of TRIF-dependent signaling pathways, did not affect the expression of SLC25A33 (Fig. 3B-D). Next, PMs were treated with an inhibitor of either PI3K or TAK1, constituents of two separate signaling pathways downstream of MyD88. We observed that the PI3K inhibitor, LY294002, significantly attenuated the protein and mRNA levels of SLC25A33 in M1 macrophages, whereas the TAK1 inhibitor, Takinib, did not (Fig. 3E-F). Consistent with the gene expression data, protein expression of SLC25A33 was decreased in LY294002-treated M1 macrophages (Fig. 3G). Considering that mTORC1 mediates ATF4 activation 29, 30, we hypothesized that PI3K/mTORC1 signaling was responsible for regulating SLC25A33 expression via ATF4 during M1 macrophage polarization. Upon treatment of LPS/IFN-γ-treated PMs with either LY294002 or rapamycin, we noted a significant attenuation in ATF4 expression, which correlated with decreased protein and mRNA levels of SLC25A33 (Fig. 3H and I). Using CD14+ monocytes isolated from septic patients, we observed that treatment with ST2825, LY294002, or rapamycin reduced SLC25A33 protein levels, mirroring the results obtained using mouse PMs (Fig. S3A and B). Furthermore, siRNA-mediated knockdown of *MyD88, PI3K*, and *mTOR*, but not *TAK1* or *TBK1,* resulted in decreased levels of ATF4 and SLC25A33 proteins, underscoring the role of MyD88-PI3K-mTORC1 signaling in the regulation of ATF4 and SLC25A33 in LPS/IFN-γ-stimulated PMs (Fig. 3J). When *ATF4* was silenced, the LPS/IFN-γ-induced upregulation of SLC25A33 was diminished at both the mRNA and protein levels, along with mRNA levels of *iNOS* and pro-inflammatory cytokines including *TNF-α*, *IL-6*, and *IL-1β* (Fig. 3K-M, and S3C). Using the Gaussia luciferase (GLuc) promoter assay, we showed that LPS/IFN-γ treatment significantly increased luciferase activity driven by the *SLC25A33* promoter in PMs transfected with a reporter plasmid containing the GLuc gene, and that *ATF4* silencing markedly decreased this activity (Fig. 3N). To determine whether ATF4 directly interacts with the promoter of *SLC25A33*, we performed chromatin immunoprecipitation (ChIP) assays using LPS/IFN-γ-treated macrophages, which revealed that the *SLC25A33* promoter region was present in significantly higher amounts in ATF4 immunoprecipitates than in IgG control immunoprecipitates (Fig. 3O). Moreover, mutations introduced into the putative ATF4 binding site within the *SLC25A33* promoter resulted in decreased LPS/IFN-γ-induced luciferase activity in PMs, when compared with cells with the intact ATF4 binding promoter vector (Fig. 3P). Collectively, these findings demonstrate that ATF4 activation through the MyD88-dependent PI3K/mTORC1 pathway plays a crucial role in regulating SLC25A33 expression during M1 macrophage polarization.

### Inhibition of SLC25A33 attenuates the cGAS-STING pathway in M1 macrophages by preventing mitochondrial DNA release

**Figure 4:**
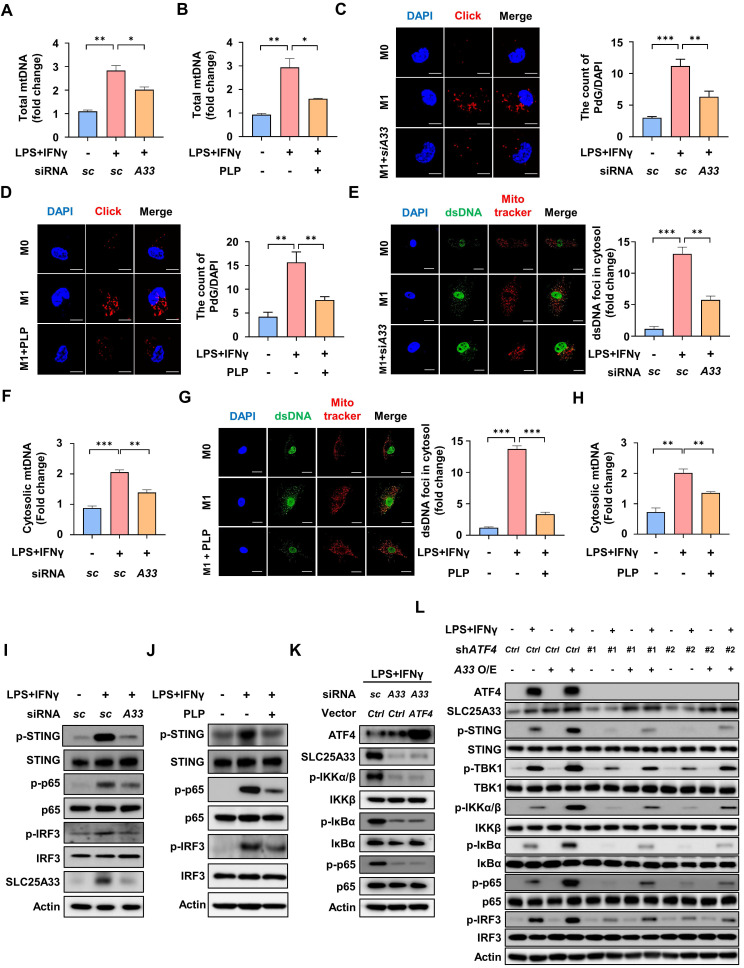
** SLC25A33 inhibition reduces mitochondrial DNA release and modulates the cGAS-STING pathway in M1 macrophages. (A-B)** Effects of *SLC25A33*-targeting siRNA (A) and PLP (B) on relative mtDNA levels in LPS/IFN-γ-treated PMs. **(C-D)** Representative immunofluorescence images using 6-*O*-Propynyl-dG (PdG) and click chemistry (left panel) and quantification of PdG (right panel) in the LPS/IFN-γ-treated PMs following treatment with *SLC25A33*-targeting siRNA (C) and PLP (D). **(E)** Representative immunofluorescence images of dsDNA and mitochondria (left panel) and quantification of cytosolic dsDNA foci (right panel) in the LPS/IFN-γ-treated PMs with *SLC25A33*-targeting siRNA. **(F)** Effect of *SLC25A33*-targeting siRNA on relative cytosolic mtDNA levels in LPS/IFN-γ-treated PMs, measured by qPCR. **(G)** Representative immunofluorescence images of dsDNA and mitochondria (left panel) and quantification of cytosolic dsDNA foci (right panel) in the LPS/IFN-γ-treated PMs with PLP.** (H)** Effect of PLP on the relative cytosolic mtDNA levels in LPS/IFN-γ-treated PMs, measured by qPCR. **(I-J)** Effects of *SLC25A33*-targeting siRNA (I) and PLP (J) on phosphorylation status of STING, p65, and IRF3 in the LPS/IFN-γ-treated PMs. **(K)** Effect of ATF4 overexpression on protein levels of SLC25A33 and phosphorylation status of IKKα/β, IκBα, and p65 in *SLC25A33* knock-downed LPS/IFN-γ-treated PMs. **(L)** Effect of SLC25A33 overexpression on phosphorylation of cGAS-STING pathway proteins in *ATF4* knock-downed LPS/IFN-γ-treated PMs. In all experiments, PMs were treated with LPS (100 ng/mL) and IFN-γ (10 ng/mL) for 24 h, with PLP (400 μM, 24 h), MitoTracker (250 nM, 30 minutes), PdG (10 μM, 1 h), and siRNAs against each gene (40 pM, 48 h). All experimental data were verified in at least three independent experiments. Data are presented as the mean ± SEM. Scale bar represents 10 μm. *p < 0.05, **p < 0.01, and ***p < 0.001. dsDNA: double-stranded DNA; PLP: pyridoxal 5'-phosphate; IRF3: interferon resgulatory factor 3; STING: stimulator of interferon genes.

Previous studies have demonstrated that transcription of mtDNA is enhanced during M1 macrophage polarization 31, 32. Consistent with these findings, our data show that LPS/IFN-γ-stimulated PMs exhibited a significant increase in total mtDNA content compared with unstimulated PMs (Fig. 4A and B). To further assess mtDNA replication, we employed 6-O-Propynyl-dG (PdG) incorporation followed by click chemistry labeling, which specifically labels newly synthesized mtDNA 33. The PdG labeling revealed a marked increase in mtDNA replication in LPS/IFN-γ-stimulated PMs (Fig. 4C and D). Interestingly, both the total mtDNA content and mtDNA replication were significantly reduced upon targeted knockdown of *SLC25A33* or PLP treatment (Fig. 4A-D). We further verified the cytosolic localization of mtDNA in our experimental conditions using double immunofluorescence (IF) staining with an anti-dsDNA antibody and MitoTracker (Fig. 4E and G). Additionally, LPS/IFN-γ-induced increases in cytosolic mtDNA were confirmed using quantitative PCR, further supporting the results showing mtDNA release into the cytosol. Notably, a significant reduction in cytosolic mtDNA was also observed following *SLC25A33* knockdown or PLP treatment (Fig. 4E-H). Cytosolic dsDNA in LPS/IFN-γ-stimulated PMs and CD14+ monocytes isolated from the septic patients was more abundant than in unstimulated PMs and its level was significantly reduced after knockdown of *SLC25A33* or treatment with PLP (Fig. S4A and B). Furthermore, we observed that LPS/IFN-γ stimulation of these cells increased the activation of the cGAS-STING pathway, as evidenced by the phosphorylation of STING, NF-κB, and IRF3, which was attenuated by both *SLC25A33* knockdown and PLP treatment (Fig. 4I-J and Fig. S4C and D). EMSA revealed that the LPS/IFN-γ-induced increase in NF-κB DNA-binding activity was attenuated by *SLC25A33* knockdown (Fig. S4E), further confirming the involvement of SLC25A33 in NF-κB activation. Notably, *SLC25A33* knockdown reduced phosphorylation of IKKα/β, IκBα, and p65 under LPS/IFN-γ treatment conditions, which persisted even after ATF4 overexpression in *SLC25A3*3 knockdown cells (Fig. 4K). By contrast, SLC25A33 overexpression increased LPS/IFN-γ-induced phosphorylated levels of STING, TBK1, IKKα/β, IκBα, p65, and IRF3 (Fig. 4L). Furthermore, the phosphorylated levels of STING, TBK1, IKKα/β, IκBα, p65, and IRF3 were higher in *ATF4* knockdown cells with SLC25A33 overexpression than in *ATF4* knockdown cells without SLC25A33 overexpression (Fig. 4L), suggesting that SLC25A33 plays a crucial role in NF-κB activation, potentially linking ATF4 to STING-TBK1-NF-κB signaling. Additionally, knockdown of *SLC25A33* markedly reduced IKKβ-dependent luciferase activity in response to LPS/IFN-γ (Fig. S4F). By contrast, *IKKβ* knockdown did not influence LPS/IFN-γ-induced protein levels of SLC25A33 or ATF4, yet it decreased p-IκBα and NF-κB levels, indicating that IKKβ functions downstream of both SLC25A33 and ATF4 (Fig. S4G). We also conducted a time-course analysis of ATF4, SLC25A33, p-STING, p-IKKα/β, p-IκBα, p-p65, and p-IRF3 protein expression in macrophages following treatment with both LPS or LPS/IFN-γ. Consistent with our hypothesis, we observed that ATF4 and SLC25A33 levels increased initially, and that this was followed by subsequent increases in the p-STING level upon LPS or LPS/IFN-γ stimulation (Fig. S4H and I). While phosphorylation of IKKα/β, IκBα, and p65 was elevated at early time points, in agreement with previous findings showing LPS-induced early NF-κB activation via TLR4 34, this phosphorylation was sustained for 24 h, indicating that ATF4 and SLC25A33 activation precedes and promotes the sequential activation of p-STING and p-TBK1, which subsequently reinforces NF-κB signaling in response to LPS or LPS/IFN-γ stimulation (Fig. S4H and I). Taken together, these data suggest that SLC25A33 plays a pivotal role in sustaining cGAS-STING pathway activity in M1 macrophages via mtDNA release.

### SLC25A33 facilitates mtDNA release via VDAC1 oligomerization and mtROS production in M1 macrophages

**Figure 5:**
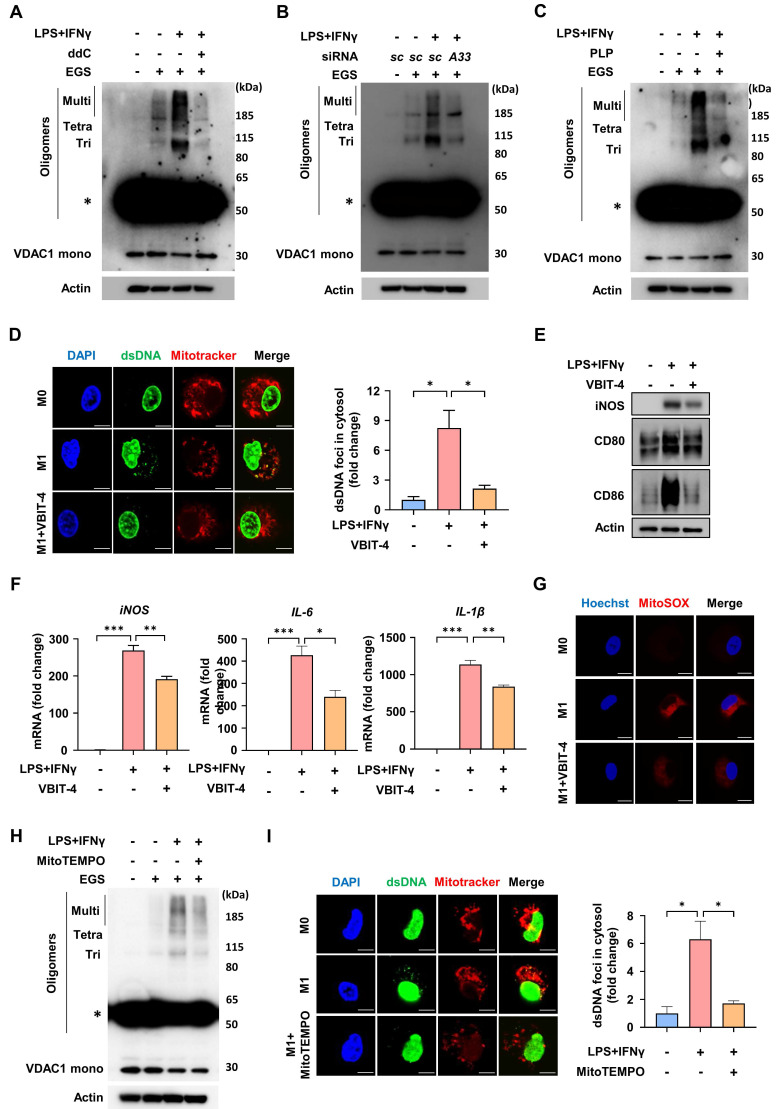
** SLC25A33 increases mtDNA release via VDAC1 oligomerization and mtROS in M1 macrophages. (A-C)** Effect of ddC (A), *SLC25A33*-targeting siRNA (B), and PLP (C) on VDAC1 oligomerization, as indicated by immunoblotting positions for monomers (Mono), trimers (Tri), tetramers (Tetra), and multimers (Multi). Asterisk indicates a nonspecific band. **(D)** Representative image of immunofluorescence staining for dsDNA and mitochondria (left panel) and quantification of cytosolic dsDNA foci (right panel) in LPS/IFN-γ-treated PMs with VBIT-4. **(E)** Effect of VBIT-4 on the protein expression level of iNOS, CD80, and CD86 in the LPS/IFNγ-treated PMs. **(F)** Effect of VBIT-4 on the mRNA expression of *iNOS* and pro-inflammatory cytokines in the LPS/IFNγ-treated PMs. **(G)** Representative image of immunofluorescence staining for MitoSOX Red in the LPS/IFN-γ-treated PMs with VBIT4. **(H)** Effect of MitoTEMPO on VDAC1 oligomerization. Asterisk indicates a nonspecific band. **(I)** Representative image of immunofluorescence staining for dsDNA and mitochondria (left panel) and quantification of cytosolic dsDNA foci (right panel) in the LPS/IFN-γ-treated PMs with MitoTEMPO. In all experiments, PMs were treated with LPS (100 ng/mL) and IFN-γ (10 ng/mL) for 24 h, with ddC (40 μM, 5 days), PLP (400 μM, 24 h), VBIT-4 (10 μM, 48 h), MitoTEMPO (500 μM, 24 h), MitoSOX Red (5 μM, 15 minutes), and *SLC25A33*-targeting siRNAs (40 pM, 48 h). All experimental data were verified in at least three independent experiments. Data are presented as the mean ± SEM. Scale bar represents 10 μm. *p < 0.05, **p < 0.01, and ***p < 0.001. ddC: 2′,3′-dideoxycytidine; dsDNA: double-stranded DNA; EGS: ethylene glycol-bis(succinimidyl succinate); VDAC1: voltage-dependent anion channel 1; PLP: pyridoxal 5'-phosphate.

The findings described above prompted us to further investigate how SLC25A33 influences mtDNA release and thereby contributes to activation of the cGAS-STING pathway in M1 macrophages. Previous studies indicated that mtDNA contributes to oligomerization of voltage-dependent anion channel 1 (VDAC1), thereby facilitating further release of mtDNA 35, 36. mtDNA depletion using 2′,3′-dideoxycytidine (ddC) reduced VDAC1 oligomerization in LPS/IFN-γ-stimulated macrophages (Fig. 5A). In concordance with these findings, both SLC25A33 knockdown and PLP treatment effectively decreased VDAC1 oligomerization in M1 macrophages (Fig. 5B-C). VBIT-4, which specifically hinders VDAC1 oligomerization irrespective of cell type 37, attenuated the LPS/IFN-γ-induced increase in cytosolic dsDNA foci (Fig. 5D). Consequently, VBIT-4 attenuated the LPS/IFN-γ-stimulated increases in iNOS, CD80, and CD86 protein expression and *iNOS*, *IL-6*, and *IL-1β* mRNA expression, indicating that mtDNA released via VDAC1 oligomers contributes to macrophage polarization (Fig. 5E-F). Interestingly, selective inhibition of VDAC1 oligomerization by VBIT-4 significantly attenuated the LPS/IFN-γ-induced increase in mtROS levels (Fig. 5G). Moreover, treatment with MitoTEMPO, a mtROS scavenger, decreased VDAC1 oligomerization, leading to reduced formation of cytosolic dsDNA foci (Fig. 5H-I). When considered together with our previous results demonstrating SLC25A33 regulation of mtROS levels, these findings indicate that SLC25A33 stimulates the release of mtDNA into the cytosol via VDAC1 oligomerization, a process that is further amplified by mtROS, which then triggers an inflammatory response.

### Reconstitution of SLC25A33-silenced BMDMs in clodronate liposome (CL) treated mice reduces inflammatory responses in LPS-induced sepsis

**Figure 6:**
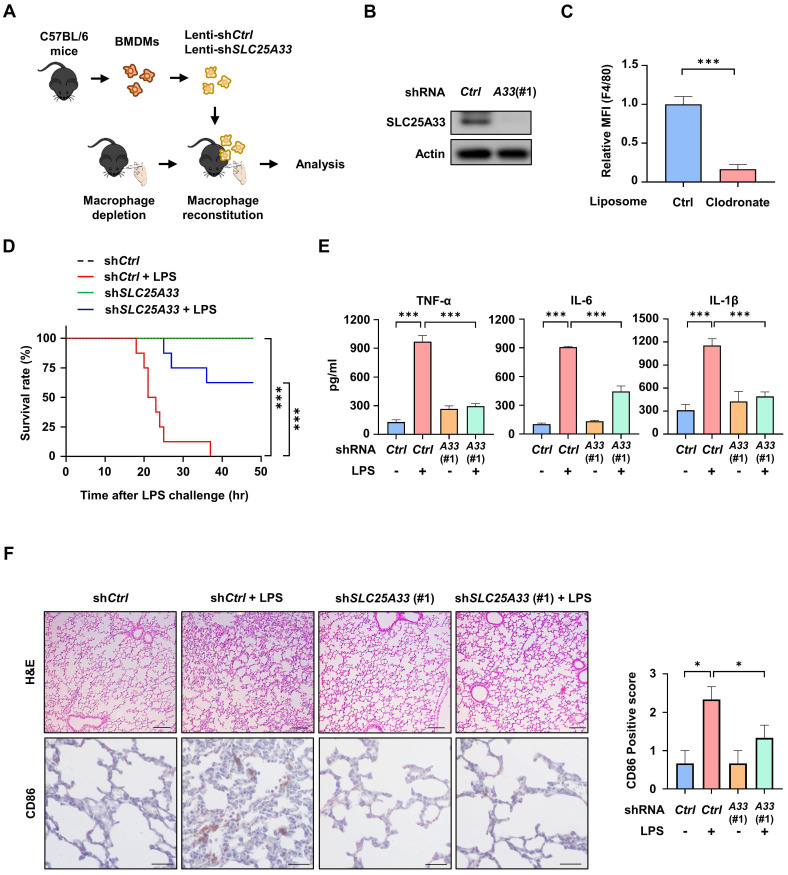
** Reconstitution with *SLC25A33*-silenced BMDMs mitigates inflammation in clodronate liposome (CL) treated mice during LPS-induced sepsis. (A)** Schematic representation of the procedure for the isolation of BMDMs from C57BL/6 male mice, transduction of *SLC25A33*-targeting shRNA, and their reconstitution into macrophage-depleted mice treated with 200 μL CL. **(B)** SLC25A33 protein levels in *SLC25A33*-silenced BMDMs before their introduction into CL-treated mice. **(C)** Quantification of macrophages in mice 2 days post CL treatment.** (D-E)** Effect of *SLC25A33*-silenced BMDMs reconstitution on the survival rate (n=8) **(D)** and pro-inflammatory cytokines (E) in LPS (30 mg/kg) induced septic mice. **(F)** Representative images of H&E staining (upper panel) (Scale bar represents 100 μm), immunohistochemical staining with anti-CD86 antibodies (lower panel) (Scale bar represents 20 μm), and the CD86 expression scores (right panel) determined using ImageJ in lung tissue sections from LPS-induced septic mice reconstituted with or without the *SLC25A33*-silenced BMDMs. All experimental data were verified in at least three independent experiments for each animal group. Data represent the mean ± SEM from independent mice. *p < 0.05, **p < 0.01, and ***p < 0.001. BMDMs: bone marrow-derived macrophages.

To determine the role of SLC25A33 in macrophages during sepsis, we depleted macrophages from mice via intravenous CL injections, before reintroducing *SLC25A33*-silenced BMDMs using three distinct clones of *SLC25A33* shRNA (Fig. 6A-B and Fig S5A). Using two distinct *SLC25A33* shRNA clones, we observed significant attenuation of LPS/IFN-γ-induced increases in the levels of SLC25A33, p-STING, p-TBK1, p-IKKα/β, p-IκBα, p-p65, and p-IRF3 in *SLC25A33* knockdown cells (Fig. S5B). These results revealed a significant decrease in the relative number of mouse spleen macrophages in the CL-treated mice compared with vehicle-treated controls (Fig. 6C). In our LPS-induced sepsis model, the CL-treated mice reconstituted with *SLC25A33*-silenced BMDMs exhibited a higher survival rate and decreased secretion of pro-inflammatory cytokines, such as TNF-α, IL-6, and IL-1β, relative to mice reconstituted with vehicle-treated BMDMs (Fig. 6D-E and S5C-D). Furthermore, these mice exhibited reduced lung injury, infiltrating inflammatory cells, and diminished CD86 expression in lung sections, as demonstrated by hematoxylin and eosin (H&E) staining and immunohistochemistry (IHC) (Fig. 6F and S5E), underscoring the potential involvement of SLC25A33 in macrophages under septic conditions. To further clarify the role of ATF4 in the *in vivo* sepsis model, we utilized *ATF4* shRNA for macrophage reconstitution in the LPS-induced sepsis mouse model. Employing two distinct *ATF4* shRNA clones, we observed that the LPS/IFN-γ-induced increases in SLC25A33, p-STING, p-TBK1, p-IKKα/β, p-IκBα, p-p65, and p-IRF3 were significantly attenuated in *ATF4* knockdown cells (Fig. S5F and G). Consistent with the findings of *SLC25A33*-silenced BMDM experiments, all CL-treated mice reconstituted with *ATF4*-silenced BMDMs exhibited markedly improved survival rates and reduced secretion of pro-inflammatory cytokines, including TNF-α, IL-6, and IL-1β, compared with mice reconstituted with vehicle-treated BMDMs (Fig. S5H and I). Additionally, H&E staining and IHC also revealed reduced infiltration of inflammatory cells and lower expression of CD86 in lung sections of mice reconstituted with *ATF4*-silenced BMDMs (Fig. S5J).

### Pyridoxal 5'-phosphate alleviates systemic inflammation in LPS-induced and cecal ligation and puncture-induced sepsis

**Figure 7:**
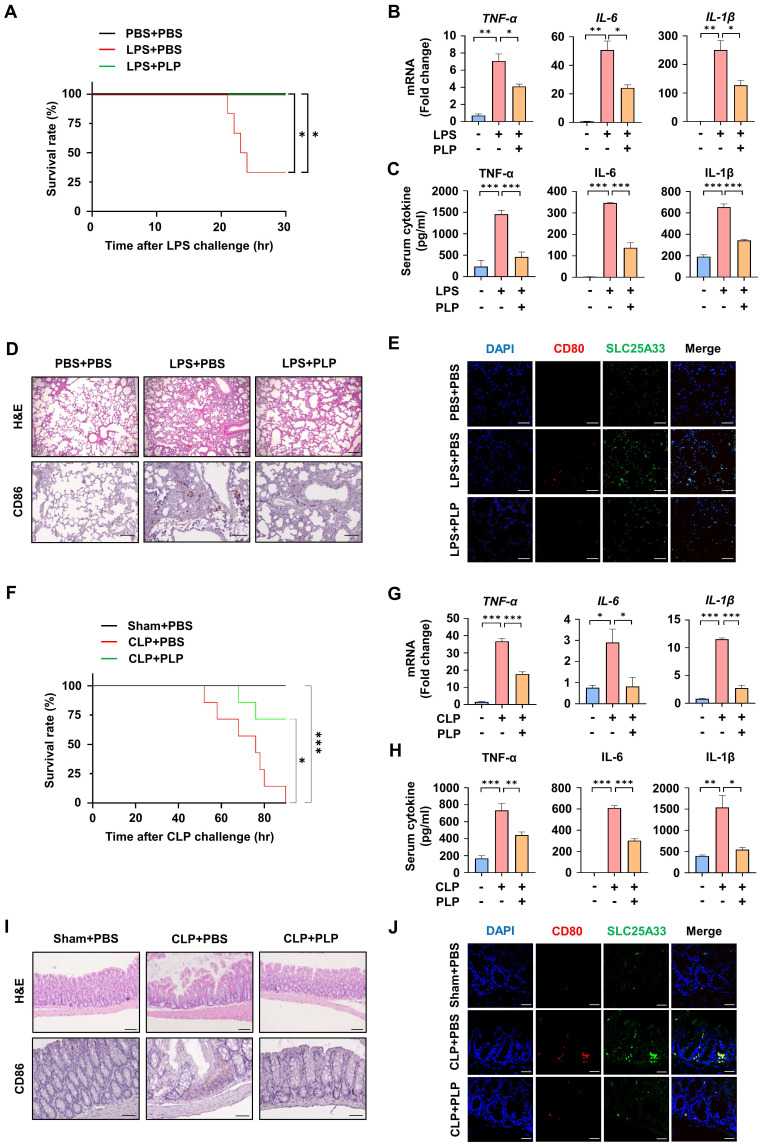
** PLP alleviates systemic inflammatory responses in LPS- and CLP-induced septic mice. (A)** Effect of PLP (20 mg/kg) on the survival rate of septic mice induced by LPS (30 mg/kg, n=6). **(B-C)** Relative mRNA levels of pro-inflammatory cytokines in PMs isolated LPS-induced (B) and their relative cytokines levels in the serum of LPS-induced (C) septic mice treated with PLP.** (D)** Representative images of H&E staining (upper panel) and immunohistochemical staining (lower panel) with antibodies against CD86 in lung tissue in the LPS-induced septic mice (Scale bar represents 100 μm). **(E)** Representative immunofluorescence images for CD80 (red) and SLC25A33 (green) in lung tissue from the LPS-induced septic mice treated with PLP (Scale bar represents 30 μm). **(F)** Effect of PLP on the survival rate of septic mice induced by CLP (n=7). **(G-H)** Relative mRNA levels of pro-inflammatory cytokines in the colon of CLP-induced (G) and their relative protein levels in the serum of CLP-induced (H) septic mice treated with PLP. **(I)** Representative images of H&E staining (upper panel) (Scale bar represents 100 μm) and immunohistochemical staining (lower panel) (Scale bar represents 40 μm) with antibodies against CD86 in colon tissue in CLP-induced septic mice. **(J)** Representative immunofluorescence images for CD80 (red) and SLC25A33 (green) in colon tissue from CLP-induced septic mice treated with PLP (Scale bar represents 40 μm). All experimental data were verified in at least three independent experiments for each animal group. Data are presented as the mean ± SEM. *p < 0.05, **p < 0.01, and ***p < 0.001. CLP: cecal ligation and puncture; PLP: pyridoxal 5'-phosphate.

Next, we investigated whether PLP could alleviate LPS- or CLP-induced systemic inflammation. When the mice were subjected to LPS-induced lethal shock, PLP-treated mice displayed better survival and lower mRNA expression, and secretion of the pro-inflammatory cytokines TNF-α, IL-6, and IL-1β, than untreated mice (Fig. 7A-C). Analysis of H&E-stained lung sections in the LPS-induced sepsis model revealed heightened histopathological changes such as alveolar septa thickening, congestion of alveolar spaces, and CD86 expression, which was significantly alleviated by PLP co-administration (Fig. 7D). This was further confirmed by IF staining, which demonstrated a PLP-induced reduction in LPS-stimulated co-localization of SLC25A33 and CD80 (Fig. 7E and S6B). Similar observations were noted in the CLP model, which more closely resembles clinical disease and human sepsis. In this model, the increase in CLP-induced mortality and pro-inflammatory cytokine production was significantly reduced following PLP treatment (Fig. 7F-H). Analysis of H&E-stained intestinal samples revealed substantial tissue destruction in the CLP-induced group (Fig. 7I), whereas administration of PLP noticeably reduced pathology and the co-localization of SLC25A33 and CD80 in IF staining (Fig. 7J and S6D). These collective findings support a pivotal role of SLC25A33 in modulating macrophage polarization and the progression of sepsis in mice.

## Discussion

In the present study, we identified SLC25A33 as a central regulatory node linking mtDNA synthesis and the inflammatory response of M1 macrophages. The study uncovers the mechanism by which SLC25A33 is upregulated in LPS/IFN-γ-stimulated macrophages and, ultimately, how SLC25A33-mediated mtDNA synthesis and attendant mtROS production contributes to M1 macrophage polarization and the activation of the cGAS-STING inflammatory pathway. The inflammatory functions of SLC25A33 were consistently observed across both *in vitro* and *in vivo* sepsis models. Importantly, increased SLC25A33 expression in blood monocytes from septic patients with liver abscesses support the significance of this protein in systemic inflammation.

Extensive research has focused on the association between individual members of the SLC25 family and inflammation 38. The roles of SLC25A37 and SLC25A22 have been previously identified, with SLC25A37 serving as an essential iron importer into the mitochondria in BMDMs, and SLC25A22 functioning as a glutamate transporter in the metabolic reprogramming of M1-like macrophages 39, 40. Although research on the mitochondrial pyrimidine transporter SLC25A33 has been limited, observations of increased pyrimidine metabolism in M1 macrophages suggest a link between pyrimidine and M1 polarization 41. Further, recent studies have demonstrated that overexpression of SLC25A33 promotes innate immunity in MEFs and HeLa cells 21. Previous work has established the role of the PI3K pathway and mTOR activity in upregulating SLC25A33 expression in transformed fibroblasts and cancer cell lines 42, 43. Our study has demonstrated that LPS stimulation induces the upregulation of SLC25A33 via MyD88-dependent PI3K and mTORC1 signaling pathways, leading to M1 polarization. Combined with our novel findings that SLC25A33 expression is driven by ATF4-dependent transcription, it appears that LPS stimulates the MyD88-PI3K-mTORC1 pathway in macrophages and that subsequent activation of ATF4 is responsible for SLC25A33 upregulation as well as M1 macrophage polarization.

Consistent with the notion that dysregulated mitochondrial nucleotide uptake and mtDNA replication may trigger the release of mtDNA into the cytosol 36, 44, we also observed that elevated SLC25A33 levels led to an increase in mtDNA synthesis and release. While various hypotheses have been proposed to explain the release of mtDNA, including alterations in mitochondrial permeability transition, changes in mitophagy, and shifts in mitochondrial dynamics, the precise mechanism remains unclear 45. Notably, VDAC1 oligomers have been identified as a critical mediator of mtDNA translocation into the cytosol, with the stabilization of VDAC1 monomer's N-terminus by mtDNA promoting oligomerization 16, 29. Consistent with these findings, our work using ddC and VBIT-4 provide further evidence supporting the role of VDAC oligomers in the cytosolic release of mtDNA. Released mtDNA triggers the cGAS-STING signaling pathway, leading to the activation of the IRF3 and NF-κB pathways, and there is evidence for a key role of this pathway in microglial polarization in CNS disorders and M1-like polarization in Brucella abortus infection 46, 47. As such, mtDNA-cGAS has been proposed as a key regulator of inflammation, orchestrating macrophage polarization towards the M1 phenotype 48. In our study, we corroborated these findings, demonstrating that LPS-induced SLC25A33 overexpression leads to augmented mtDNA synthesis and release into the cytosol, triggering cGAS-STING signaling and M1 macrophage polarization.

In line with previous studies showing that VDAC oligomerization increases mtROS production 35, 36, the findings further indicate that both SLC25A33 inhibition and VBIT-4 treatment effectively reduce mtROS levels in M1 macrophages. Intriguingly, this reduction in mtROS correlated with a decrease in VDAC oligomer and cytosolic mtDNA levels, demonstrating the existence of a positive feedback loop between VDAC oligomerization and mtROS that contributes to mtDNA release into the cytosol during M1 macrophage polarization. However, the exact mechanism via which VDAC oligomerization increases mtROS production remains unclear 49. We propose that VDAC1 oligomerization in the outer mitochondrial membrane triggers the release of cytochrome c, a potent antioxidant, into the cytosol. This release reduces the availability of cytochrome c within the mitochondria, leading to an increase in mtROS production 49. Conversely, it has been suggested that mtROS promote VDAC oligomerization by oxidizing cardiolipin, a mitochondrial membrane lipid that binds cytochrome c. When cardiolipin is oxidized, cytochrome c detaches from the membrane, which in turn can increase VDAC oligomerization 50, 51. Additionally, VDAC1 interacts with hexokinase (HK) at the outer mitochondrial membrane, which regulates mitochondrial permeability and suppresses mtROS production 52, 53. VDAC1 oligomerization could potentially disrupt this interaction with HK, reducing its inhibitory effect on mtROS generation. Further studies are needed to clarify the precise interplay between VDAC1 oligomerization and mtROS production.

We employed LPS-induced septic shock models, both with and without reconstitution with SLC25A33-silenced BMDMs, alongside a CLP-induced sepsis model, to examine the role of SLC25A33 in the inflammatory responses *in vivo*. While the LPS model is critical for studying acute systemic inflammation, the CLP model offers a more gradual timeline for the development of systemic inflammation, more closely mimicking human sepsis 54, 55. Inhibition of SLC25A33 in macrophages alleviates pro-inflammatory responses and prolongs survival in both LPS- and CLP-induced models. Furthermore, the expression of SLC25A33 in CD14+ monocytes, which correlates with pro-inflammatory cytokine production and clinical resolution in sepsis patients, underscores the clinical significance of SLC25A33 in macrophage-mediated inflammatory diseases. Given that PLP, as utilized in the experiments of this study, is not a highly specific inhibitor of SLC25A33, it remains possible that PLP exerts an inhibitory influence on inflammatory cell infiltration through mechanisms unrelated to SLC25A33. Indeed, PLP is known to modulate various metabolic and immunological processes 56, supporting this possibility. Although the substantial overlap between the effects of PLP treatment and SLC25A33 knockdown suggests that at least part of PLP's immunomodulatory activity involves SLC25A33 inhibition, future studies employing more selective inhibitors are warranted.

In conclusion, the data presented herein demonstrate that LPS-induced overexpression of SLC25A33 triggers mtDNA synthesis and cytosolic release, thereby demonstrating that SLC25A33 has a pivotal role in the inflammatory response of M1 macrophages during sepsis. These findings imply that targeting SLC25A33 could be a promising treatment strategy for managing M1 macrophage-mediated inflammatory diseases such as sepsis.

## Supplementary Material

Supplementary figures and tables.

## Abbreviations

mtDNA: mitochondrial DNA
cGAS: cyclic GMP-AMP synthase
STING: stimulator of interferon genes
IFN-I: type I interferons
IFN-γ: interferon-gamma
TLR: toll-like receptor
SLC25: solute carrier family 25
PMs: peritoneal macrophages
PBMCs: peripheral blood mononuclear cells
PdG: 6-O-Propynyl-dG
CLP: cecal ligation and puncture
BMDMs: bone marrow-derived macrophages
PLP: pyridoxal 5'-phosphate
GLuc: Gaussia luciferase
ddC: 2',3'-dideoxycytidine
VDAC1: voltage-dependent anion channel 1
