# Supplementary material for: SLC25A33-mediated mitochondrial DNA synthesis plays a critical role in the inflammatory response of M1 macrophages by contributing to mitochondrial ROS and VDAC oligomerization
Source: Int J Biol Sci. 2025 Apr 21;21(7):2935–53. doi: 10.7150/ijbs.96563 (PMC12080393; doi:10.7150/ijbs.96563)
Supplement: Supplementary file 1 — Supplementary figures and tables. [file ijbsv21p2935s1.pdf]

Supplementary figure

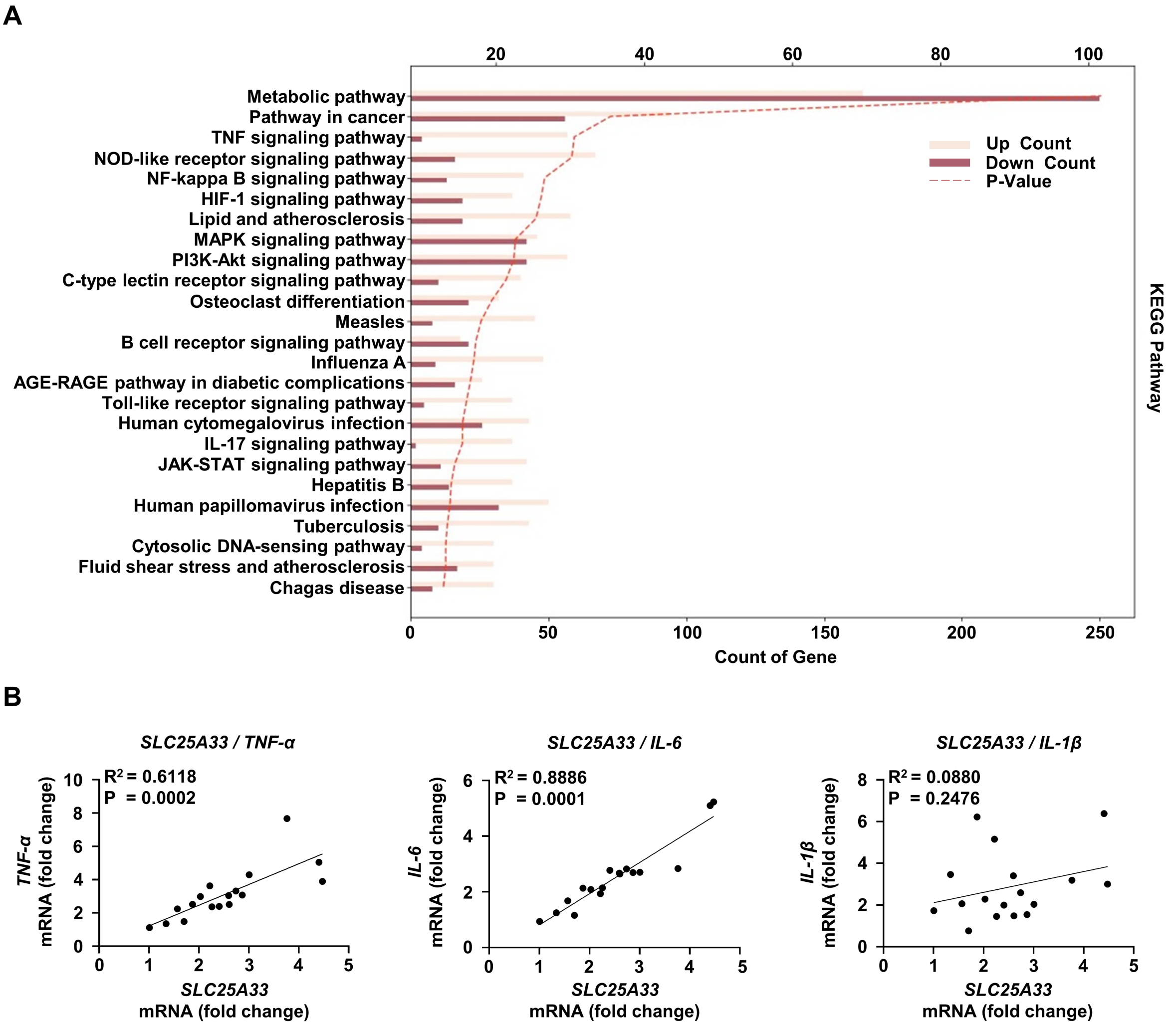

Fig. S1. KEGG pathway analysis of differentially expressed genes and correlation between fold changes in *SLC25A33* expression and fold changes in pro-inflammatory cytokine expression

(A) KEGG pathway analysis of differentially expressed genes using DAVID in LPS/IFN-γ-treated and untreated PMs. **(B)** Correlation between mRNA levels of pro-inflammatory cytokines and *SLC25A33* in CD14+ monocytes derived from patients with sepsis (n=17).

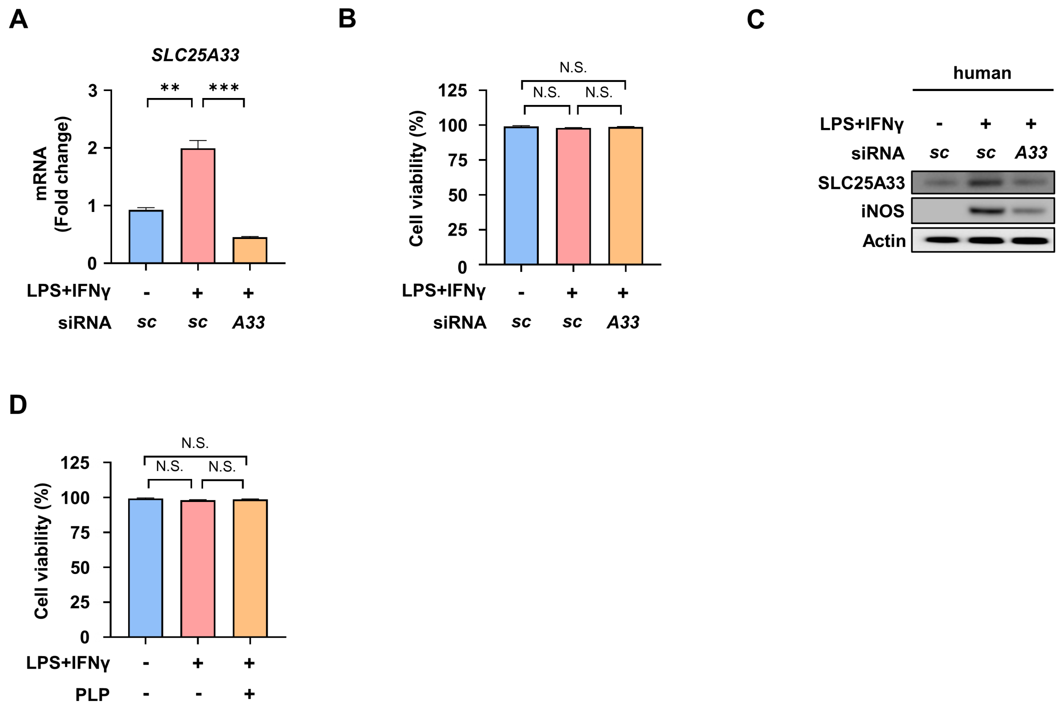

Fig. S2. SLC25A33 inhibition does not affect PM cell viability but reduces iNOS expression in human monocytes

(A) Effect of *SLC25A33*-targeting siRNA on mRNA expression of *SLC25A33* in the LPS/IFN-γ-treated PMs. **(B)** Cell viability in LPS/IFN-γ-treated PMs with or without *SLC25A33*-targeting siRNA in the LPS/IFN-γ-treated PMs. **(C)** Effect of *SLC25A33*-targeting siRNA on protein levels of SLC25A33 and iNOS in CD14+ monocytes isolated from septic patients. **(D)** Cell viability in LPS/IFN-γ-treated PMs with or without PLP. In all experiments, cells were treated with LPS (100 ng/mL) and IFN-γ (10 ng/mL) for 24 h, with PLP (400 μM, 24 h), and with *SLC25A33*-targeting siRNA (0.04 nM, 48 h). All experimental data were verified in at least three independent experiments. Data are presented as the mean ± SEM. N.S., not significant, $^{**}p < 0.01$ and $^{***}p < 0.001$.

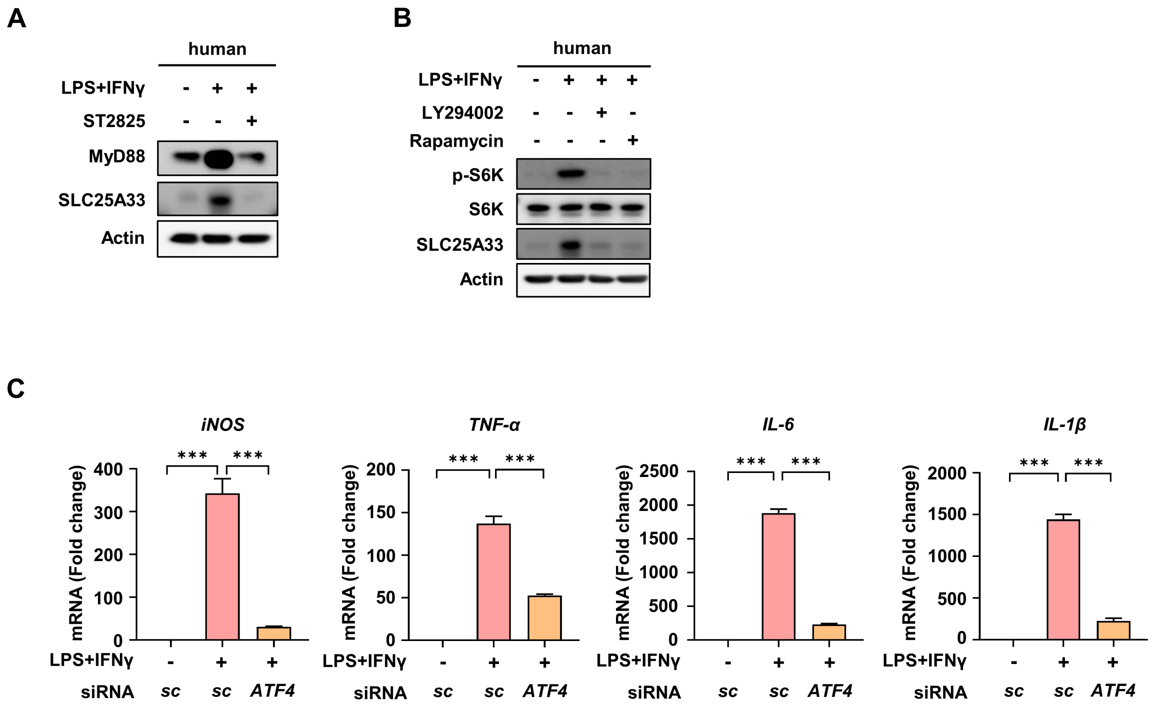

Fig. S3. Inhibition of MyD88-PI3K-mTORC1 pathway decreases SLC25A33 expression, and *ATF4* knockdown reduces the mRNA levels of pro-inflammatory markers

(A-B) Effects of ST2825 (A) and LY294002 or rapamycin (B) on the protein levels of SLC25A33 in LPS/IFN-γ-treated human CD14+ monocytes. **(C)** Effect of *ATF4*-targeting siRNA on mRNA expression of *iNOS* and pro-inflammatory cytokines in LPS/IFN-γ treated PMs. Cells were treated with LPS (100 ng/mL) and IFN-γ (10 ng/mL) for 24 h, with ST2825 (10 µM, 24 h), LY294002 (25 nM, 24 h), rapamycin (50 nM, 24 h), and *SLC25A33*-targeting siRNA (0.04 nM, 48 h). All experimental data were verified in at least three independent experiments. Data are presented as the mean ± SEM. $^{***}p < 0.001$.

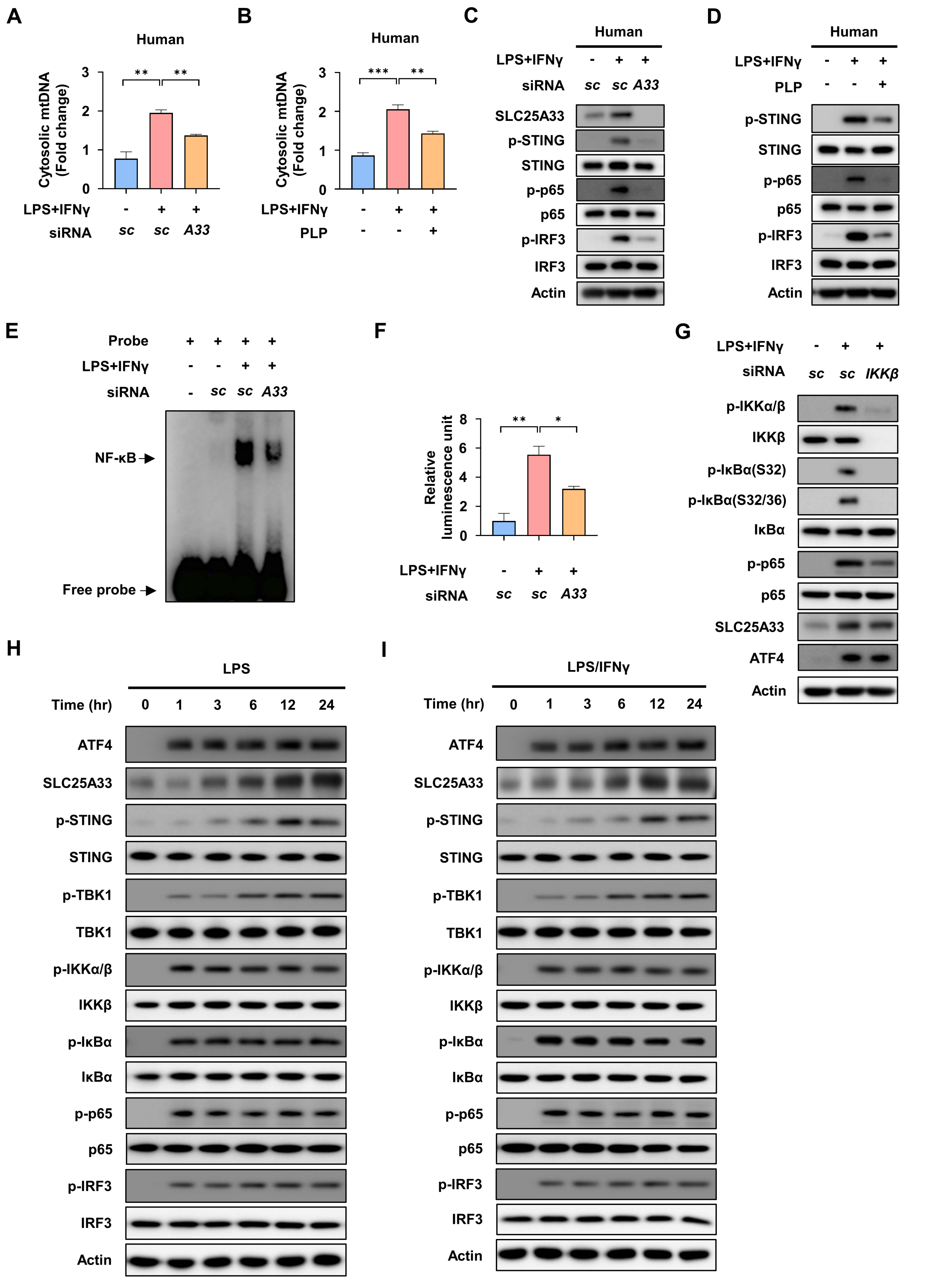

Fig. S4. Inhibition of SLC25A33 reduces the protein expression of the cGAS-STING pathway in human monocytes, while SLC25A33 activation increases NF-κB signaling in response to LPS or LPS/IFN-γ stimulation in PMs

(A-B) Effect of *SLC25A33* knockdown with siRNA (A) or PLP (B) on relative cytosolic

mtDNA levels in LPS/IFN-γ-treated human CD14+ monocytes. **(C-D)** Effect of *SLC25A33* knockdown with siRNA (C) or PLP (D) on phosphorylation status of cGAS-STING pathway proteins in LPS/IFN-γ-treated human CD14+ monocytes. **(E)** EMSA analysis of NF-κB activity in response to *SLC25A33*-targeting siRNA in the LPS/IFN-γ-treated PMs. **(F)** Effect of *SLC25A33*-targeting siRNA on IKKβ activity measured by ADP-Glo kinase assay in LPS/IFN-γ-treated PMs. **(G)** Effect of *IKKβ*-targeting siRNA on protein levels of ATF4 and SLC25A33 and phosphorylation status of IKKα/β, IκBα, and p65 in the LPS/IFN-γ-treated PMs. **(H-I)** Time-course analysis of protein levels of ATF4 and SLC25A33 and phosphorylation status of key proteins in the cGAS-STING pathway in PMs treated with LPS (H) or LPS/IFN-γ (I). In all experiments, cells were treated with LPS (100 ng/mL) and IFN-γ (10 ng/mL) for 24 h with PLP (400 μM, 24 h), and with *SLC25A33*-targeting siRNA (0.04 nM, 48 h). All experimental data were verified in at least three independent experiments. Data are presented as the mean ± SEM. $^*p < 0.05$, $^{**}p < 0.01$, $^{***}p < 0.001$.

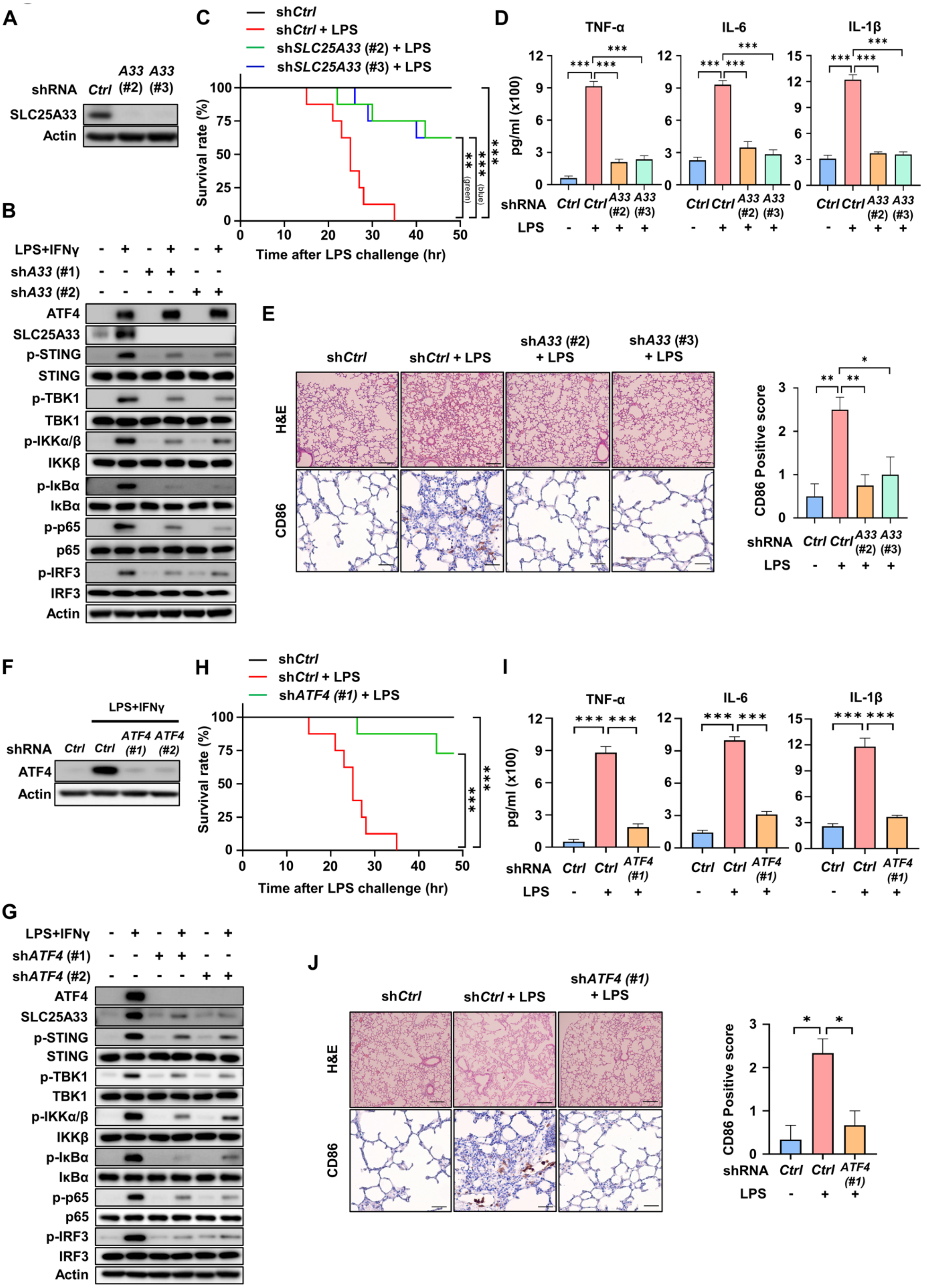

Fig. S5. Reconstitution with *SLC25A33*- or *ATF4*- silenced BMDMs attenuates inflammation in clodronate liposome (CL) treated mice during LPS-induced sepsis

(A) SLC25A33 protein levels in two differently silenced *SLC25A33* BMDM groups prior to

their introduction into CL-treated (200 μL) mice. **(B)** Effect of LPS/IFN-γ treatment on protein level of ATF4 and phosphorylation status of key proteins in the cGAS-STING pathway in sh*SLC25A33* PMs. **(C-D)** Impact of each reconstituted *SLC25A33*-silenced BMDM on survival rates (n=8) (C) and pro-inflammatory cytokine levels (D). **(E)** Representative images of H&E staining (upper panel, scale bar: 100 μm), immunohistochemical staining with anti-CD86 antibodies (lower panel, scale bar: 20 μm), and CD86 expression scores (right panel) determined using ImageJ in lung tissue sections from LPS (30 mg/kg)-induced septic mice, reconstituted with or without each *SLC25A33*-silenced BMDM. **(F)** ATF4 protein levels in *ATF*4-silenced BMDMs before their introduction into CL-treated mice. **(G)** Effect of LPS/IFN-γ treatment on protein level of SLC25A33 and phosphorylation statuses of key proteins in cGAS-STING pathway in sh*ATF4* PMs. **(H-I)** Effect of reconstituted *SLC25A33*-silenced BMDMs on survival rates (n=8) (H) and pro-inflammatory cytokine levels (I). **(J)** Representative images of H&E staining (upper panel, scale bar: 100 μm), immunohistochemical staining with anti-CD86 antibodies (lower panel, scale bar: 20 μm), and CD86 expression scores (right panel) determined using ImageJ of lung tissue sections from LPS-induced septic mice, reconstituted with and without each *ATF4*-silenced BMDM. In all experiments, cells were treated with LPS (100 ng/mL) and IFN-γ (10 ng/mL) for 24 h. All experimental were data verified in at least three independent experiments for each animal group. Data are presented as the mean ± SEM. **p < 0.01, ***p < 0.001.

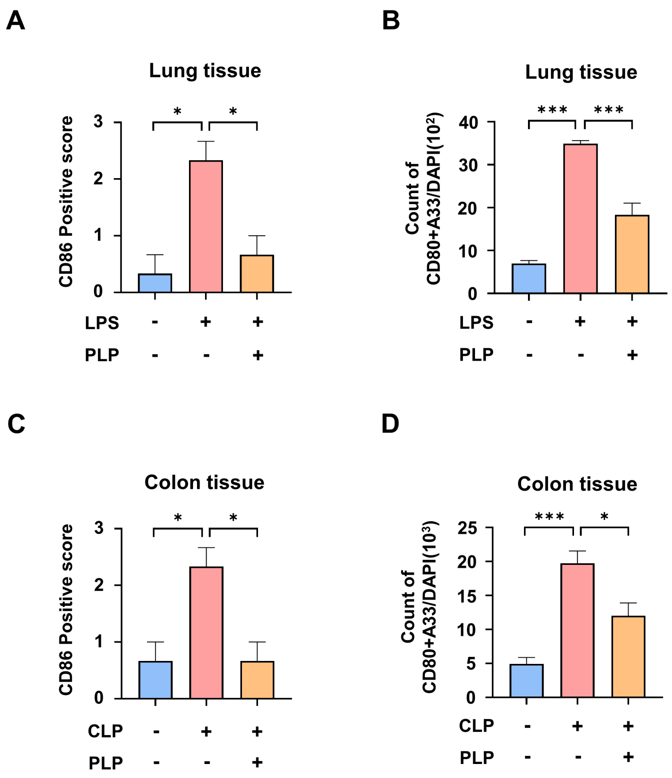

Fig. S6. PLP mitigates inflammatory responses in septic mice.

(A-B) CD86 expression scores (A) and quantification of co-localized CD80 and SLC25A33 expression relative to DAPI-stained nuclei (B) in lung tissue sections from the LPS (30 mg/kg)-treated septic mice, with or without PLP (20 mg/kg). **(C-D)** CD86 expression scores (C) and quantification of co-localized CD80 and SLC25A33 expression relative to DAPI-stained nuclei (D) in colon tissue sections from the CLP model, with or without PLP (20 mg/kg). All experimental data were verified in at least three independent experiments for each animal group. Data are presented as the mean ± SEM. $*p < 0.05$, $***p < 0.001$. CLP, cecal ligation and puncture; PLP, pyridoxal 5'-phosphate.

Supplementary table

Table S1 Top 1,000 up- and down-regulated genes in LPS/IFN-γ-treated PMs

Differential expression analysis was performed to identify genes with significant changes. Genes belonging to the solute carrier 25 family are highlighted in bold.

	Gene name
**Up-regulated genes**	1110032F04Rik, 1110038F14Rik, 1600014C10Rik, 1700003M07Rik, 1700034I23Rik, 1700123I01Rik, 1810009A15Rik, 1810037I17Rik, 1810055G02Rik, 2010005H15Rik, 2210039B01Rik, 2210416O15Rik, 2310043L19Rik, 2500002B13Rik, 2700054A10Rik, 3110057O12Rik, 3110062M04Rik, 4921511I17Rik, 4930440I19Rik, 4930599N23Rik, 4933412E12Rik, 4933416M07Rik, 4933432I03Rik, 4933433H22Rik, 6430562O15Rik, 6530402F18Rik, 8430430B14Rik, 9330175E14Rik, 9330179D12Rik, 9430037G07Rik, 9430076C15Rik, 9630013K17Rik, A230028O05Rik, A430093F15Rik, A530032D15Rik, A630012P03Rik, A730020E08Rik, AA467197, Abca13, Abhd16a, Abhd2, Abracl, Abtb2, Acot7, Acpp, Acsl1, Acvrl1, Adarb2, Adcy6, Adgb, Adgra2, Adgrf3, Adm, Adora2a, Adora2b, Adora3, Agpat5, Agtrap, Ahr, AI504432, AI854517, Aida, Ak2, Ak4, Akt3, Alas1, Alkbh2, Alpk2, Amica1, Ankrd37, Ankrd66, Apba3, Apobec3, Apol10b, Apol6, Apol9a, Apol9b, Appbp2os, Aqp9, Arf5, Arg1, Arhgap28, Arhgap8, Arhgef3, Arhgef37, Arid5a, Arl1, Arl4a, Arl5c, Arnt2, Arrdc4, Art3, Asap3, Ascl2, Asns, Atp1b3, AW011738, AW112010, B3gnt5, Batf, Batf2, BC048507, BC094916, BC147527, Bcl2a1a, Bcl2a1b, Bcl2a1d, Bcl2l14, Best1, Birc3, Blvrb, Bnip3, Brd2, Bst1, Bst2, Btg3, C130026I21Rik, C1ra, C1rb, C1rl, C1s1, C1s2, C3, C9, Cabp4, Cacybp, Camp, Car13, Car2, Casp1, Casp12, Casp4, Casp7, Ccdc25, Ccdc58, Ccdc85b, Ccdc88b, Ccl12, Ccl17, Ccl2, Ccl22, Ccl3, Ccl5, Ccl7, Ccl8, Ccnd2, Ccne1, Ccr7, Ccrl2, Cd14, Cd1d1, Cd1d2, Cd200, Cd274, Cd300e, Cd38, Cd40, Cd52, Cd69, Cd83, Cd86, Cdc42ep2, Cdk2ap2, Cdkn1a, Ceacam18, Cebpb, Cenpj, Cept1, Cers6, Cfb, Cflar, Ch25h, Chac1, Chic1, Chil4, Chrna1, Ciart, Ciita, Cish, Cks2, Clcf1, Clec2d, Clec4e, Clec4n, Clec5a, Clic4, Clic5, Clmp, Clrn3, Cmpk2, Cnih4, Cnn3, Coa5, Coq10b, Cox17, Cp, Creb5, Crem, Csf3, Csrnp1, Cst7, Ctgf, Ctrl, Ctsc, Cxcl1, Cxcl10, Cxcl11, Cxcl16, Cxcl2, Cxcl3, Cxcl5, Cxcl9, Cycs, Cysltr2, Cytip, D16Ertd472e, D330045A20Rik, D6Ertd527e, D730005E14Rik, Daxx, Dck, Dcstamp, Ddit4, Ddit4l, Ddx43, Dennd3, Denr, Dgat2, Dgkh, Dhx58, Dnajb3, Dnase1l3, Dpys, Dram1, Draxin, Dst, Dtd1, Dtx2, Dtx3l, Dusp16, Dusp28, Dvl3, Ebi3, Edn1, Efcab2, Egln1, Egln3, Ehd1, Eif4ebp1, Elavl2, Emb, Emc9, Endou, Eno1b, Eno1b, Enpp4, Epha1, Erap1, Ereg, Ero1l, Ets1, Eva1b, Exoc3l4, F10, F3, F630111L10Rik, F830016B08Rik, Fam102b, Fam114a1, Fam13a, Fam162a, Fam26f, Fam65b, Fam71f2, Fanca, Fas, Fbxl5, Fcgr1, Fcgr2b, Fcgr4, Fcrla, Ffar2, Fgl2, Fhl3, Filip1l, Flnb, Flt4, Fndc7, Fosl1, Foxp4, Fpr1, Fpr2, Fzd1, G530011O06Rik, Gabrr2, Gadd45b, Gbgt1, Gbp10, Gbp11, Gbp2, Gbp2b, Gbp3, Gbp4, Gbp5, Gbp6, Gbp7, Gbp8, Gbp9, Gca, Gch1, Gdap10, Gfpt1, Ggct, Gimap9, Gja1, Glipr2, Glrx, Gm10684, Gm10872, Gm11110, Gm11127, Gm12185, Gm12216, Gm12250, Gm13251, Gm13498, Gm14005, Gm14023, Gm14085, Gm14379, Gm15056, Gm15133, Gm15645, Gm15987, Gm16675, Gm16712, Gm1720, Gm17757, Gm18853, Gm1966, Gm19705, Gm4070, Gm4841, Gm4907, Gm4951, Gm4961, Gm5069, Gm5177, Gm5424, Gm5431, Gm5483, Gm5523, Gm614, Gm6524, Gm6642, Gm6682, Gm6904, Gm6981, Gm7030, Gm8369, Gm8979, Gm8989, Gm9895, Gm9992, Gmfg, Gna13, Gnaz, Gnb4, Gng12, Gosr1, Gpd2, Gphb5, Gpm6b, Gpn2, Gpr132, Gpr141, Gpr171, Gpr18, Gpr35, Gpr84, Gpr85, Grap, Grasp, Grhpr, Grina, Gsap, Gsr, Gstt1, Gstt4, Gtf2e2, Gtpbp2, Gvin1, Gxylt2, Gyk, Gys1, H2-Ab1, H2-DMa, H2-M2, H2-M3, H2-Oa, H2-Q4, H2-Q5, H2-Q7, H2-Q8, H2-Q9, H2-T10, H2-T22, H2-T9, Hamp, Has1, Hat1, Hbegf, Hcar2, Hck, Hcls1, Hdc, Helz2, Herc6, Hid1, Hif1a, Higd1a, Hilpda, Hk2, Hk3, Hmox1, Hrc, Hrh2, Hsp90aa1, Hspa1a, Hspa1b, Hspa1l, Hspa4l, Hspa8, Htr7, Htra4, I830077J02Rik, Icam1, Idnk, Ier3, Ifi203, Ifi204, Ifi205, Ifi35, Ifi44, Ifi47, Ifih1, Ifit1, Ifit1bl1, Ifit1bl2, Ifit2, Ifit3, Ifit3b, Ifitm1, Ifitm3, Ifng, Igf2bp1, Igfbp3, Igtp, Iigp1, Il10, Il12a, Il12b, Il12rb1, Il13ra1, Il15, Il15ra, Il18bp, Il18rap, Il1a, Il1b, Il1f6, Il1f9, Il1rn, Il23a, Il23r, Il27, Il2ra, Il2rg, Il6, Il7, Ildr1, Inhba, Inpp5b, Insl6, Intu, Irak3, Irf1, Irf7, Irf8, Irg1, Irgm1, Irgm2, Isca1, Isg15, Isg20, Ism1, Isoc1, Itga1, Itgal, Itprip, Jag1, Jak2, Jam2, Jdp2, Jmjd6, Junb, Kalrn, Katna1, Kctd12, Kdm4b, Kdm6b, Klk9, Klkb1, Klra2, Klrk1, Kynu, Lacc1, Lad1, Lap3, Lck, Lcn2, Lcp2, Ldha, Leng9, Lhx2, Lif, Lipg, Lipn, Lmo1, Lmo4, Lpar1, Lpar4, Lpcat2, Lrp11, Lrrc16a, Lrrc2, Lrrc4, Lrrc75a, Lrrc8c, Lrrk2, Lta, Ly6a, Ly6c1, Ly6c2, Ly6f, Ly6i, Lysmd2, Lztfl1, M6pr, Mab21l3, Maff, Mag, Magohb, Malt1, Map2k1, Map4, 1.Mar, 5.Mar, Marcksl1, Marco, Max, Mcemp1, Mefv, Met, Mfsd7a, Mgarp, Micall2, Mif, Mip, Mlkl, Mmp13, Mmp2, Mmp25, Mnda, Mndal, Mov10, Ms4a4c, Ms4a4d, Ms4a6b, Ms4a6c, Ms4a6d, Msantd3, Mst1r, Mt2, Mtfr2, Mthfd1l, Mthfd2, Mx1, Mx2, Mxd1, Mybpc3, Mycl, Myd88, Mylpf, Naa25, Nampt, Ncr1, Ndrg1, Net1, Nfil3, Nfkb1, Nfkb2, Nfkbia, Nfkbib, Nfkbie, Nfkbiz, Ninj1, Nlrc5, Nlrp3, Nmi, Nod1, Nod2, Nos2, Nr4a3, Nrg1, Nrp2, Nrxn3, Nt5c3, Nupr1, Nxpe3, Oasl1, Oasl2, Odc1, Ogfr, Olfr56, Olr1, Orai2, Osgin2, Osm, Otud1, P2ry10, P2ry14, P4ha1, P4ha2, Pacsin1, Papolb, Parp10, Parp11, Parp12, Parp14, Parp8, Parp9, Pax4, Pbld1, Pcgf5, Pcp4, Pdcd1, Pdcd1lg2, Pde10a, Pde11a, Pde4b, Pde5a, Pdk1, Pdpn, Pdxp, Pecam1, Peli1, Perm1, Pfkfb3, Pfkl, Pfkp, Pgk1, Pgm2, Phf11a, Phf11b, Phf11c, Phf11d, Phlda1, Phlpp1, Pi4k2b, Pilra, Pilrb1, Pilrb2, Pim1, Pim2, Pim3, Pkp4, Pla1a, Pla2g16, Pla2g4a, Pla2g7, Plac8, Plaur, Plbd1, Plek, Plekhg4, Pnp, Pnp2, Pola2, Polr2d, Pomp, Pou3f1, Ppa1, Ppap2a, Ppap2b, Ppfia3, Ppm1n, Ppp1r15a, Ppp1r15b, Ppp1r26, Prdx1, Prdx5, Prdx6b, Prelid2, Prm1, Procr, Prr15, Psat1, Psma4, Psma5, Psma6, Psma7, Psmb10, Psmb8, Psmb9, Psmd10, Psme1, Psme2, Psme2b, Pstpip2, Ptcra, Ptges, Ptgs2, Ptgs2os, Ptgs2os2, Ptpn2, Ptx3, Pvr, Pydc3, Pydc4, Pyhin1, Rab11fip1, Rab32, Rab33a, Rab43, Rac2, Ralgds, Ramp3, Ran, Rap2c, Rapgef2, Rasgrp1, Rbbp8, Rbl1, Rbpms, Rcl1, Rcn1, Relb, Rfx5, Rgcc, Rgs11, Rgs9, Rhbdf2, Rhoh, Ripk2, Rmdn3, Rmi2, Rnase10, Rnaseh2b, Rnaset2a, Rnaset2b, Rnd1, Rnd3, Rnf114, Rnf144a, Rnf19b, Rnf217, Rnf225, Rsad2, Rtn2, Rtn4r, Runx3, Ruvbl2, Ryr2, S100pbp, Saa3, Samhd1, Samsn1, Sav1, Scarf1, Scarletltr, Scimp, Sdc4, Sdcbp2, Seh1l, Sell, Sema4a, Sema7a, Sepw1, Serf1, Serpina3f, Serpina3g, Serpina3h, Serpinb1a, Serpinb1b, Serpinb2, Serpinb6b, Serpinb9, Serpine1, Serping1, Sfmbt2, Sfrp1,

	Sh3bp4, Siglece, Slamf1, Slamf6, Slamf7, Slamf8, Slc15a3, Slc16a1, Slc16a3, **Slc25a22, Slc25a33, Slc25a37, Slc25a43**, Slc28a2, Slc2a1, Slc2a6, Slc31a1, Slc31a2, Slc32a1, Slc4a11, Slc5a10, Slc6a19, Slc7a11, Slc7a2, Slc7a3, Slco3a1, Slfn1, Slfn2, Slfn4, Slfn8, Slfn9, Slx4ip, Smpdl3b, Snhg8, Snx10, Socs1, Socs3, Sod2, Sod3, Sorbs1, Sox5, Sp110, Sp140, Spaca6, Spata13, Spic, Sppl2a, Sprtn, Src, Srgn, St3gal5, St7, Stat1, Stat2, Stat3, Stat4, Stc2, Stfa2l1, Stfa3, Stx11, Stx2, Stxbp3, Styk1, Syt7, Tacstd2, Tagap, Tank, Tap1, Tap2, Tapbp, Tapbpl, Tarm1, Tbl2, Tcp10a, Tcp10b, Tcp10c, Tefm, Tescl, Tgm1, Tgm2, Tgtp1, Tgtp2, Thbs1, Ticam2, Timd4, Timp1, Tiparp, Tjp1, Tjp3, Tlr6, Tlr9, Tm2d1, Tma16, Tmbim4, Tmeff1, Tmem128, Tmem132a, Tmem140, Tmem170b, Tmem171, Tmem178, Tmem2, Tmem200c, Tmem202, Tmem243, Tmem42, Tmem44, Tmem67, Tmigd1, Tmprss9, Tmsb4x, Tmtc2, Tnf, Tnfaip2, Tnfaip3, Tnfaip8l3, Tnfrsf11b, Tnfrsf1b, Tnfrsf8, Tnfsf10, Tnfsf14, Tnfsf15, Tnfsf9, Tnip1, Tnip3, Tnp2, Tor3a, Tpi1, Tppp3, Tpst1, Traf1, Traf2, Trafd1, Trem1, Trem3, Treml2, Trex1, Trim12a, Trim12c, Trim21, Trim30a, Trim30b, Trim30d, Trim6, Tspan3, Tspo, Ttc39b, Ttc39c, Ttc9c, Tulp2, Tvp23a, Twsg1, Txlnb, Txn1, Txnrd1, Tyms-ps, U90926, Uba7, Ubd, Ube2l6, Ugcg, Unc5a, Upp1, Usb1, Usf1, Usp18, Vasp, Vcam1, Vcan, Vegfa, Vhl, Vnn3, Wars, Wasf3, Wdr20rt, Wfdc17, Wfdc18, Whamm, Wnk2, Wnt6, Xaf1, Xcr1, Xkr8, Zadh2, Zbp1, Zbtb32, Zbtb5, Zc3h12a, Zc3h12c, Zc3hav1l, Zcwpw1, Zdhhc2, Zfp558, Zfp800, Zfp811, Zfp819, Zmynd15, Zyx
**Down-regulated genes**	Gm29811, 0610040J01Rik, 1110019D14Rik, 1110051M20Rik, 1190005I06Rik, 1500026H17Rik, 1600010M07Rik, 1700017B05Rik, 1700026L06Rik, 1700102P08Rik, 1810011H11Rik, 1810034E14Rik, 2210011C24Rik, 2410004P03Rik, 2610035D17Rik, 2810025M15Rik, 2900008C10Rik, 3110021A11Rik, 4632428N05Rik, 4930412C18Rik, 4930556M19Rik, 4930565N06Rik, 4931403E22Rik, 4933400F21Rik, 4933404O12Rik, 5031425F14Rik, 5430427M07Rik, 5430431A17Rik, 5830416I19Rik, 5930430L01Rik, 6430548M08Rik, 8430419L09Rik, 9030617O03Rik, 9130019P16Rik, 9530077C05Rik, 9830147E19Rik, 9930012K11Rik, A230056J06Rik, A330032B11Rik, A430088P11Rik, A930019D19Rik, A930024E05Rik, AA388235, AA415398, Aatk, Abca2, Abca9, Abcb4, Abcb6, Abcc3, Abcd2, Abcg2, Abcg3, Abhd12, Abhd15, Acaa2, Acaca, Acad10, Acap3, Accs, Acot2, Acrbp, Acsf2, Acsl3, Adam12, Adamts10, Adat1, Adcy7, Adcy9, Add3, Adgre5, Adgrl1, Adk, Adssl1, Agap1, Agbl3, Ahdc1, AI839979, Ak8, Akr7a5, Aldh2, Aldh9a1, Alox5, Alox5ap, Ang, Angpt2, Angptl2, Angptl4, Ank, Ankrd13b, Ankrd23, Ankrd55, Anxa3, Ap1s2, Apbb1, Aph1c, Apoc2, Apoc4, Apoc4-apoc2, Arap2, Arap3, Arhgap15, Arhgap18, Arhgap22, Arhgap24, Arhgap39, Arhgap6, Arhgef18, Arhgef40, Arhgef6, Arl11, Arsg, Asap1, Asb4, Aspm, Asrgl1, Atg9b, Atp13a2, Atp1a3, Atp2a3, Atp6v0a1, Atp6v0d2, Atxn1, AU022793, B130034C11Rik, B230217C12Rik, B3galnt1, B3glct, B3gnt7, B3gnt8, B4galnt1, B4galt4, B4galt6, B630019K06Rik, Bach2, Bach2os, Bahcc1, Baiap2l1, Bbs9, BC100451, Bcar3, Bcl2, Bcl2l11, Bcl7a, Bex1, Bfsp1, Bmf, Bmp2, Bmx, Bmyc, Bok, Brca2, Bsn, Btbd17, Bzw2, C030034L19Rik, C130036L24Rik, C330006A16Rik, C5ar1, C5ar2, C77080, Cacna2d4, Calcoco1, Calhm2, Camk1, Camk4, Capn9, Car11, Car5b, Card11, Card14, Casc1, Catsperg1, Cbfa2t3, Cbr2, Cbx6, Ccdc15, Ccdc152, Ccdc176, Ccdc69, Ccpg1, Ccsap, Cd109, Cd200r1, Cd24a, Cd28, Cd300a, Cd300lb, Cd300lg, Cd59a, Cd5l, Cd93, Cd99l2, Cda, Cdan1, Cdc25b, Cdc42ep1, Cdc7, Cdca3, Cdca7, Cdca7l, Cdk18, Cdk20, Cdkn1c, Cdkn3, Ceacam1, Ceacam2, Cebpa, Cela1, Celf4, Cenpe, Cep131, Cercam, Cerk, Cfh, Cgnl1, Chd3, Chek2, Chn2, Chp2, Chst14, Cib2, Ckap4, Ckb, Clcn5, Clec10a, Clec2i, Cln6, Clvs1, Cmbl, Cnn1, Cnnm2, Cnnm3, Cnr2, Cnrip1, Col14a1, Col19a1, Col1a1, Colec12, Cotl1, Cpb2, Cpeb3, Cr2, Cracr2b, Cradd, Creb3l1, Crygn, Cryl1, Csad, Csrnp2, Cstad, Ctns, Ctsd, Cx3cr1, Cxcl14, Cxcr3, Cxcr4, Cxx1a, Cxx1b, Cxx1c, Cyp27a1, Cyp2j6, Cyp51, D2hgdh, D8Ertd82e, Dab2, Dach1, Dagla, Daglb, Dchs1, Dcxr, Dear1, Depdc7, Deptor, Des, Dgkd, Dgkg, Dhcr24, Dhcr7, Dhrs3, Dio2, Dlg2, Dmrta2, Dnah11, Dnajb13, Dnajc9, Dnm1, Dnmt3aos, Dock1, Dock10, Dock8, Dpep3, Dppa3, Dtx3, Dtx4, Dusp9, E2f2, Ebpl, Edaradd, Eef2k, Eepd1, Efna5, Efr3b, Ehd4, Emc8-1190005i06rik, Emp1, Engase, Enpp1, Enpp3, Epas1, Epb4.1l1, Eps8, Esco2, Espl1, Etv5, Exo1, F13a1, F2rl2, F5, Fabp4, Fabp5, Fads1, Fam102a, Fam105a, Fam110b, Fam117a, Fam120c, Fam132a, Fam134b, Fam161a, Fam171a1, Fam189b, Fam196a, Fam198b, Fam20a, Fam213a, Fam214a, Fam229b, Fam53b, Fam57a, Fam65a, Fam78a, Fam84b, Fancd2os, Fasn, Fbln2, Fbn2, Fbxo32, Fbxw10, Fcgrt, Fcrl1, Fcrls, Fdft1, Fgd2, Fgd4, Fgfr1, Fggy, Fhdc1, Fhl2, Fli1, Flrt1, Fmnl1, Fmo5, Fn1, Foxd2os, Foxred2, Frat1, Frat2, Fry, Fsbp, Fut10, Fut7, Fzd3, G730013B05Rik, Gab3, Galc, Galm, Galnt12, Galnt9, Gamt, Gas1, Gas6, Gata6, Gatsl2, Gcat, Gcnt1, Gdf3, Gdpd5, Gfod1, Gfra2, Ggt5, Glul, Gm10336, Gm10389, Gm10638, Gm10825, Gm13375, Gm13710, Gm14461, Gm15706, Gm15708, Gm16287, Gm1673, Gm16907, Gm20300, Gm20605, Gm4788, Gm4890, Gm5086, Gm5547, Gm9920, Gmpr, Gng7, Gpd1l, Gpr155, Gpr157, Gpr160, Gpr162, Gpr183, Gpr82, Gpx1, Gpx3, Grk4, Gsn, Gstm7, Gtf2i, Guca2b, H19, H60b, Habp4, Hacd4, Hadh, Hal, Hao1, Havcr2, Hdac5, Hebp1, Hey1, Hfe, Hgf, Hhex, Hip1, Hmha1, Hnmt, Hoxb3, Hpgds, Hsd17b14, Hsf3, Hspa12a, Htra3, Id3, Idh1, Idh2, Ier5l, Ifi202b, Ifi27, Ifngr1, Igf1, Igf1r, Il16, Il1r1, Inf2, Inpp4b, Insr, Iqgap2, Iqsec1, Irf4, Irs2, Itga6, Itgb3, Itgb3bp, Itgb5, Jmjd1c, Jph3, Kank2, Katnb1, Kcnk6, Kcnq1, Kcnq1ot1, Kctd12b, Kdelc2, Kdsr, Khk, Kif13b, Kif14, Kif23, Kif5a, Kifc3, Klc4, L1cam, Large, Ldhb, Ldlrad3, Lefty1, Lfng, Lima1, Lincppara, Lipa, Lmbrd2, Lmna, Lmtk3, Lpar6, Lpin1, Lpl, Lppr2, Lpxn, Lrmp, Lrp1, Lrp4, Lrp6, Lrrc20, Lrrc27, Lrrc34, Ltc4s, Lyl1, Lyzl4, Macrod1, Mafb, Mak, Mamdc2, Maml2, Maml3, Man1c1, Man2a2, Man2b1, Maoa, Map3k9, Map6, Mapre3, Mark4, Marveld1, Mast3, Matk, Matn2, Mbd4, Mblac2, Mbnl3, Mcam, Mccc2, Mcm2, Mcoln3, Mctp1, Mdga1, Mef2a, Mef2c, Megf9, Melk, Metrn, Mettl7a1, Mfge8, Mfsd6, Mgat5, Mgmt, Mical1, Mical3, Mid1ip1, Mknk2, Mmp27, Mospd3, Mr1, Mrc1, Mrgpre, Msh2, Msrb2, Mtap7d3, Mtfp1, Mtmr11, Mtss1, Mum1l1, Mutyh, Mvb12b, Mxd4, Myh10, Mylk4, Myom1, Mzt2, Nacc2, Nanos1, Nat8l, Nav1, Nav2, Nbeal2, Ncapg, Ncapg2, Nceh1, Nckap5l, Nckipsd, Ncor2, Ndst1, Nedd9, Neil2, Neil3, Neurl1b, Neurl2, Nfatc1, Nfia, Nfic, Nhsl2, Nicn1, Nim1k, Ninl, Nipal3, Nipsnap1, Nkain1, Nlrc3, Nlrp10, Nlrp1b, Nlrp1c-ps, Nme3, Nme4, Nos3, Nostrin, Npas2, Npas4, Nqo2, Nrep, Nrm, Nrp1, Nt5dc2, Nuak1, Nup210, Nusap1, Nxpe4, Nxpe5, Nynrin, Oaz3, Ogg1, Oit3, Olfml3, Olfr172, Ophn1, Osbpl6, P2rx7, P2ry1, Padi2, Padi3, Padi4, Pak3, Pald1, Palm, Palm3, Pam, Pank1, Paox, Paqr7, Pawr, Pbx1, Pcbp3, Pccb, Pcgf2, Pcp4l1, Pcyox1l, Pcyt1b, Pdcd4, Pde4a, Pdgfa, Pdgfc, Pdk2, Pdxk, Pdzd4, Pecr, Peg13, Peli2, Penk, Per3, Pex11g, Pfas, Pfkfb4, Pgm2l1, Pgm5, Phka2, Phlda3, Pianp, Piezo2, Pif1, Pigb, Pigz, Pink1, Pkib, Pla2g15, Pla2g2e, Plekhf1, Plekhg3, Plk1, Plscr4, Plxdc1, Plxna2, Plxna4, Plxnc1, Plxnd1, Pnoc, Pnpla7, Pola1, Pomgnt2, Porcn, Ppapdc3, Pparg, Ppfia4, Ppm1j, Ppp1r32, Ppp1r3fos, Pram1, Prc1, Prkag2, Prkg1, Prokr1, Prps2, Prss46, Prtn3, Prune2, Psd3, Ptchd1, Pter, Ptgfrn, Ptgs1, Ptk7, Ptpn22, Ptprs, Pvt1, Pwwp2b, Pygb, Pyroxd2, Rab11fip4, Rab11fip5, Rab3il1, Rab40c, Rab42, Rab4a, Rab6b, Rac3, Rad9b, Ramp1, Raph1, Rapsn, Rasa3, Rasal2, Rasgrf2, Rasgrp2, Rasgrp3, Rassf2, Rassf5, Rcbtb2, Rcsd1, Rdm1, Rell1,

	Rem1, Reps2, Retnlg, Retsat, Rfng, Rfx2, Rgl2, Rgs10, Rgs18, Rgs2, Rhbdl1, Rhob, Rian, Rin1, Rin3, Rmrp, Rnase4, Rnf144b, Rnf150, Rnf180, Rpp40, Rrad, Rttn, Rxra, S100a4, S100g, S1pr1, S1pr4, Sap25, Sash3, Sat2, Sbk1, Sc5d, Scamp5, Scarna3b, Scd1, Scfd2, Sel1l3, Sepn1, Sepp1, Serac1, Serinc5, Serpinb6a, Sesn1, Sgol2a, Sgsh, Sh2d1b1, Sh2d3c, Sh3bgrl2, Sh3pxd2a, Shb, Shc4, Sipa1l2, Ski, Slc16a2, Slc16a4, Slc16a7, Slc16a9, Slc17a5, Slc17a9, Slc19a1, Slc1a5, Slc24a1, **Slc25a23, Slc25a24**, Slc26a11, Slc29a1, Slc2a8, Slc35c2, Slc36a1, Slc36a2, Slc37a2, Slc39a5, Slc43a2, Slc46a1, Slc46a3, Slc7a15, Slc9a3r2, Slc9a9, Slco2b1, Smagp, Smarca2, Smim6, Smpd3, Smyd3, Snai2, Snora75, Snord92, Snta1, Snx24, Snx29, Snx30, Snx32, Soga1, Sorbs3, Sort1, Sox7, Sox9, Spag17, Specc1, Spib, Sprr2a2, Spry2, Sqle, Srd5a1, Ssbp3, Ssh2, Sspn, St6gal1, St8sia4, Stard4, Steap3, Stk32c, Stmn1, Ston2, Stra6l, Stxbp4, Sulf2, Sult2b1, Suox, Susd3, Suv420h2, Svip, Syne1, Syne3, Syngr1, Synj2, Synpo, Syt11, Tacc2, Tarsl2, Tbc1d16, Tcp11l2, Tecpr1, Tef, Tenm4, Tfap4, Tfdp2, Tgfbr1, Thbd, Thnsl2, Thsd1, Timp2, Tk1, Tle1, Tle6, Tlr13, Tmcc1, Tmem114, Tmem119, Tmem135, Tmem144, Tmem14a, Tmem154, Tmem158, Tmem176a, Tmem176b, Tmem180, Tmem191c, Tmem238, Tmem254a, Tmem254b, Tmem254c, Tmem26, Tmem35, Tmem37, Tmem41a, Tmem64, Tmem65, Tmem71, Tmem8, Tmem86a, Tmsb15b1, Tnfrsf11a, Tnfrsf22, Tnfsf12, Tnfsf13, Tnfsfm13, Tnk2, Tnni2, Tns1, Tns4, Tpcn1, Tpk1, Tpmt, Trem2, Treml1, Trerf1, Trf, Trib1, Trib2, Trim2, Trim29, Trim47, Trim68, Troap, Trp53inp1, Trpv4, Tsc22d3, Tst, Ttc28, Ttc7, Ttk, Ttyh2, Tulp4, Txndc16, Ubald1, Ube2d2b, Uckl1os, Ulk2, Umps, Unc119, Unc13a, Usp2, Usp20, Utp14b, Vars2, Vegfb, Vps13c, Vsig10l, Vsig4, Vwf, Wbscr27, Wdfy4, Wdr7, Wnt4, Wwc2, Wwp1, Xdh, Xylt1, Ydjc, Ypel2, Ypel3, Zak, Zbtb16, Zbtb20, Zbtb38, Zdhhc14, Zfhx2, Zfhx3, Zfp219, Zfp296, Zfp362, Zfp36l1, Zfp385a, Zfp395, Zfp493, Zfp608, Zfp667, Zfp704, Zfp808, Zfp882, Zfpm1, Zfyve28, Znrf3

Table S2. ***SLC25A33*** **gene expression levels in PMs, with or without LPS/IFN-γ treatment**

Expression data were derived from Digital Gene Expression analysis and processed using the quantile normalization method via EdgeR. The table shows data from three independent biological replicates for each condition.

	M0 (1)	M0 (2)	M0 (3)	M1 (1)	M1 (2)	M1 (3)
SLC25A33	**35.24**	**58.00**	**37.98**	**133.62**	**152.32**	**140.95**

Table S3. The characteristics of septic patients with liver abscess.

Patient ID	Age	Sex	SLC25A33		TNF-α		IL-6		IL-1β		CRP (mg/dL)		PCT (ng/mL)	WBC (count/μl)		PLT (count/μl)		GOT (U/L)		GPT (U/L)		SIRS
			Pre	Post	Pre	Post	Pre	Post	Pre	Post	Pre	Post		Pre	Post	Pre	Post	Pre	Post	Pre	Post	
Patient 1	65	M	2.03	0.47	2.99	0.57	2.08	0.55	2.28	0.46	19.50	0.07	6.95	24680	6210	225k	169k	94	40	42	13	4
Patient 2	79	F	1.57	0.59	2.25	1.12	1.68	0.64	2.06	0.25	24.16	0.06	21.38	19890	8750	175k	433k	185	23	128	17	3
Patient 3	57	F	2.87	1.67	3.08	1.42	2.69	1.77	1.55	0.80	13.80	0.45	0.20	15740	3890	403k	177k	31	45	25	19	3
Patient 4	75	M	1.87	0.99	2.52	1.11	2.13	0.89	6.23	0.84	21.82	0.12	51.30	5210	6280	47k	232k	69	23	62	10	2
Patient 5	68	M	2.74	1.07	3.32	2.59	2.82	0.88	2.59	3.95	35.71	0.61	9.89	10200	7940	92k	264k	54	22	19	5	4
Patient 6	89	F	2.26	1.11	2.37	1.74	2.14	0.95	1.45	0.95	12.77	0.09	0.36	12990	3600	420k	360k	56	29	36	14	2
Patient 7	22	F	2.22	1.58	3.63	1.69	1.93	1.22	5.16	2.28	17.02	0.06	0.74	13040	3370	424k	629k	25	45	16	44	4
Patient 8	60	F	4.41	2.31	5.04	3.12	5.10	1.90	6.39	5.47	13.84	0.07	0.34	17650	6990	203k	260k	98	32	85	56	3
Patient 9	73	M	2.41	-	2.39	-	2.77	-	1.99	-	12.79	0.40	5.70	6880	4650	123k	415k	98	18	70	21	3
Patient 10	62	F	1.70	-	1.49	-	1.16	-	0.76	-	16.91	-	-	10040	-	126k	-	93	-	216	-	2
Patient 11	78	F	1.01	-	1.12	-	0.94	-	1.73	-	21.43	-	1.59	16310	-	243k	-	33	-	47	-	3
Patient 12	63	F	1.34	-	1.35	-	1.25	-	3.46	-	6.00	-	0.35	25670	-	590k	-	26	-	23	-	3
Patient 13	91	F	2.61	-	2.51	-	2.64	-	1.49	-	30.73	-	2.23	30230	-	157k	-	228	-	152	-	4
Patient 14	83	F	3.77	-	7.77	-	2.84	-	3.19	-	8.36	-	-	14280	-	357k	-	69	-	27	-	3
Patient 15	52	M	2.60	-	3.04	-	2.67	-	3.40	-	22.83	-	6.02	13640	-	204k	-	77	-	73	-	4
Patient 16	72	F	3.77	-	7.67	-	2.84	-	3.20	-	30.22	-	1.25	15920	-	288k	-	43	-	29	-	4
Patient 17	75	M	4.48	-	3.90	-	5.23	-	3.00	-	10.83	-	0.18	7000	-	167k	-	37	-	36	-	2

CRP: C-reactive protein; GOT: glutamic oxaloacetic transaminase; GPT: glutamic pyruvic transaminase; PLT: platelet; PCT: procalcitonin; SIRS: systemic inflammatory response syndrome. SIRS criteria is defined when at least two of the following criteria are met: a body temperature above 38 or below 36 degrees Celsius, a heart rate exceeding 90 beats per minute, a respiratory rate over 20 breaths per minute or a partial pressure of CO_2 below 32 mmHg, and a leukocyte count above 12,000 or below 4,000 per microliter or over 10% immature forms or bands.

Table S4. The characteristics of healthy controls.

Patient ID	Age	Sex	SLC25A33	TNF-α	IL-6	IL-1β
Healthy 1	40	F	1.00	1.00	1.00	1.00
Healthy 2	30	M	1.53	1.35	1.44	1.72
Healthy 3	31	M	0.06	0.07	0.06	0.13
Healthy 4	37	M	0.13	0.10	0.08	0.19
Healthy 5	30	M	0.26	0.27	0.24	0.26
Healthy 6	27	M	0.16	0.14	0.16	0.30
Healthy 7	27	F	0.73	0.63	0.58	0.76
Healthy 8	30	F	0.40	0.38	0.35	0.37
Healthy 9	37	F	0.88	0.80	0.71	1.26
Healthy 10	52	M	0.77	0.67	0.62	0.72

The relative expression levels of SLC25A33, TNF-α, IL-6, and IL-1β are represented relative to healthy control 1.
